# SOCS3 Suppression Promoted the Recruitment of CD11b^+^Gr-1^−^F4/80^−^MHCII^−^ Early-Stage Myeloid-Derived Suppressor Cells and Accelerated Interleukin-6-Related Tumor Invasion *via* Affecting Myeloid Differentiation in Breast Cancer

**DOI:** 10.3389/fimmu.2018.01699

**Published:** 2018-07-23

**Authors:** Wenwen Zhang, Mengmeng Jiang, Jieying Chen, Rui Zhang, Yingnan Ye, Pengpeng Liu, Wenwen Yu, Jinpu Yu

**Affiliations:** ^1^Cancer Molecular Diagnostics Core, Tianjin Medical University Cancer Institute and Hospital, National Clinical Research Center for Caner, Key Laboratory of Cancer Prevention and Therapy, Tianjin’s Clinical Research Center for Cancer, Tianjin, China; ^2^Department of Urology, Tianjin Medical University General Hospital, Tianjin, China; ^3^Department of Immunology, Tianjin Medical University Cancer Institute and Hospital, National Clinical Research Center for Caner, Key Laboratory of Cancer Immunology and Biotherapy, Tianjin’s Clinical Research Center for Cancer, Tianjin, China

**Keywords:** breast cancer, interleukin-6, early-stage myeloid-derived suppressor cells, SOCS3, the JAK/STAT signal pathway, myeloid differentiation

## Abstract

Interleukin-6 (IL-6) is an important trigger for the expansion and recruitment of myeloid-derived suppressor cells (MDSCs), which are regarded to be major coordinators of the immunosuppressive tumor microenvironment. In this study, we constructed IL-6-knockdown breast cancer mice models to explore the molecular events involved in the IL-6-mediated effects on MDSC development. We defined a subset of early-stage MDSCs (e-MDSCs) with the phenotype of CD11b^+^Gr-1^−^F4/80^−^MHCII^−^ in IL-6 high-expressing 4T1 mice mammary carcinoma models, which were the precursors of CD11b^+^Gr-1^+^ conventional MDSCs. Furthermore, sustained suppression of SOCS3 and aberrant hyperactivation of the JAK/STAT signaling pathway was exclusively detected in wide-type 4T1 tumor-bearing mice, which promoted the accumulation of e-MDSCs *in situ* and their immunosuppressive capability *in vitro*. After blocking the IL-6/STAT3 signaling pathway with the IL-6 receptor antibody or STAT3 antagonist JSI-124 in tumor-bearing mice, significant shrinkage of primary tumors and decrease in lung metastatic nodules were observed *in vivo*, accompanied by the dramatic decrease of e-MDSC recruitment and recovery of anti-tumor T cell immunity. Thus, SOCS3 suppression accelerated the IL-6-mediated growth and metastasis of mammary carcinoma *via* affecting myeloid differentiation in breast cancer. Moreover, the IL-6/STAT3 signaling pathway might be a promising candidate target in developing novel therapeutic strategies to eliminate e-MDSCs and improve breast cancer prognosis.

## Introduction

Immune suppression is a major barrier to effective cancer immunotherapy, and the accumulation of myeloid-derived suppressor cells (MDSCs) has recently been recognized as a major mechanism that promotes immune suppression. MDSCs are a heterogeneous population of immature myeloid cells found within tumor microenvironments that exert suppressive effects on both innate and adaptive immunity (1–3). However, their phenotypes in cancer are rather diverse. MDSCs in mice are defined by the expression of CD11b and Gr-1. In mice CD11b^+^ cells, two main MDSC populations have been characterized: monocytic MDSCs (M-MDSCs, CD11b^+^Ly6G^−^Ly6C^hi^) and polymorphonuclear MDSCs (PMN-MDSCs, CD11b^+^Ly6G^hi^Ly6C^−^) (4). In humans, similar cell subsets (M-MDSCs and PMN-MDSCs) were detected with distinct phenotypes of CD11b^+^HLA-DR^−/lo^CD14^+^CD15^−^ and CD11b^+^HLA-DR^−/lo^CD14^−^CD15^+^, respectively (5). Recently, “early-stage MDSCs” (e-MDSCs) with the phenotype of Lin^−^HLA-DR^−^CD33^+^, which comprise more immature progenitors, have been defined as the third subset of human MDSCs (6). However, its counterpart in mice and the development of e-MDSCs have still not been disclosed.

In our previous study, we identified a subset of immature MDSCs in human breast cancer tissues with the phenotype of Lin^−^HLA^−^DR^−^CD45^+^CD33^+^CD13^+^CD14^−^CD15^−^ that exerted potent immunosuppressive effects on T cells *in vitro* and *in vivo* (7). These primary MDSCs significantly correlated with advanced clinical stage, higher lymph node metastasis, and poor prognosis (7, 8), which indicated that these immature MDSCs were representatives of e-MDSCs in breast cancer. Furthermore, we found positive correlation between the level of tumor-derived interleukin-6 (IL-6) and the recruitment of e-MDSCs locally (9). IL-6 potently promoted the amplification of e-MDSCs and their T cell-suppressive capacity *in vitro* by activating the STAT/IDO signaling pathway and generating a tryptophan-starved microenvironment that facilitated the evasion of breast cancer cells (8, 9). Our previous study also demonstrated that tumor-derived IL-6 might play a significant role in the development and accumulation of e-MDSCs *in vivo*; however, the regulatory mechanisms underlying IL-6-related myeloid differentiation blockage are less understood.

Interleukin-6 is a pleiotropic cytokine with significant functions in the regulation of pro-inflammatory and metastatic tumor microenvironments (10). Accumulating evidences showed significant correlation between IL-6 and MDSC development in human and murine models (11–13). It is well established that IL-6 activates tyrosine kinases Janus kinase 1 (JAK1), Janus kinase 2 (JAK2), and tyrosine kinase 2 (TYK2) *via* IL-6 receptor (IL-6R) and gp130, which leads to the phosphorylation of signal transducers and activators of transcriptions 1 and 3 (STAT1 and STAT3) (14, 15). IL-6-dependent activation of the JAK/STAT signaling pathway is tightly regulated by members of the suppressor of cytokine signaling (SOCS) protein family (16), and quick feedback of SOCS1/SOCS3 upregulation efficiently inhibits the phosphorylation of STAT3 under physiologic conditions, thereby attenuates the activation of the JAK/STAT signaling pathway and expression of downstream functional genes (17, 18).

However, sustained activation of the JAK/STAT signaling pathway was observed in breast cancer e-MDSCs because of significant SOCS3 suppression, which consequently induced the long-term activation of the NF-κB signaling pathway and suppression of T cell immunity (9). STAT3 has been reported to be essential in maintaining a well-differentiated and fully competent immune system (14). Therefore, SOCS3 deficiency-dependent sustained activation of the JAK/STAT signaling pathway might regulate the differentiation of myeloid progenitors. Multiple hemopoietic and immunological defects were also reported in SOCS1/SOCS3-deficient mice as a consequence of prolonged STAT3 activation (19–21). Croker et al. found that the differentiation of the SOCS3-deficient progenitor cells skewed toward macrophage production due to poor response to G-CSF (22). Furthermore, Yu et al. found that SOCS3 deletion in myeloid cells produced higher levels of CD11b^+^Gr-1^+^ MDSCs in prostate tumors (23). Therefore, it will be essential to clarify that if SOCS3 deficiency and sustained activation of the JAK/STAT signaling pathway blocked the differentiation of myeloid progenitors and thus promoted e-MDSC development in breast cancer.

In this study, we constructed IL-6-knockdown 4T1 murine mammary carcinoma-bearing models to study the effects of tumor-derived IL-6 on the development of e-MDSCs *in vivo* to determine whether SOCS3 deficiency and sustained activation of the JAK/STAT signaling pathway blocked the differentiation of myeloid linkage and promoted the recruitment of e-MDSCs locally. We defined a subset of e-MDSCs with a poorly differentiated phenotype of CD11b^+^Gr-1^−^F4/80^−^MHCII^−^ in mice mammary carcinoma, which were the precursors of CD11b^+^Gr-1^+^ conventional MDSCs and exerted more potent suppression on T cell immunity. Tumor-derived IL-6 impaired the differentiation of myeloid cells and promoted the accumulation of e-MDSCs by inhibiting SOCS3 expression and persistently activating the JAK/STAT signaling pathway. Moreover, IL-6R blocking antibody and STAT3 antagonist JSI-124 effectively inhibited the growth of primary tumors and distance metastases in lungs while simultaneously reducing the recruitment of e-MDSCs *in situ* and reversing T cell immunosuppression *in vitro*. Thus, we concluded that the IL-6-related dysfunction of the SOCS3 feedback loop accelerated the growth and metastasis of mammary carcinoma by affecting myeloid differentiation and attenuating the T cell-based immune surveillance. Furthermore, the IL-6/STAT3 signaling pathway could be a promising candidate target in developing novel therapeutic strategies to eliminate e-MDSCs and improve breast cancer prognosis.

## Materials and Methods

### Cell Lines and Mice

BALB/c female mice were purchased from Beijing Vital River Laboratory Animal Technology, and 6- to 8-week-old mice were used in the experiments. The mouse mammary tumor cell line (4T1) used in this study was purchased from the Chinese Academy of Medical Sciences and cultured in RPMI-1640 medium (Gibco/BRL, Grand Island, NY, USA) containing 10% fetal bovine serum (FBS) in a humidified 5% CO_2_ incubator at 37°C. By transfecting wild-type 4T1 cells (4T1^WT^) with IL-6-specific short hairpin RNA (shRNA) lentiviral vectors, 4T1^IL-6low^ cells were established as indicated in the Supplementary data 1 in Supplementary Material. Nonsense lentiviral shRNA vector-transfected 4T1^WT cell^s were established as 4T1^NC^ controls. All transfected cells were screened using 3 µg/mL of puromycin (Gibco, Grand Island, NY, USA). Stably transfected cells were cloned by limiting dilution and validated using RT-PCR and enzyme-linked immunosorbent assay (ELISA) before expansion. And anti-IL-6R mAb (10 µg/mL; Bio X Cell, USA) was utilized to block IL-6 signaling in 4T1^IL-6low^ cells *in vitro*.

### Construction of 4T1 Breast Cancer-Bearing Murine Models

Cells (4T1^WT^, 4T1^NC^, or 4T1^IL-6low^; 1 × 10^6^ cells in 200 µL PBS) were injected into the mammary fat pads of BALB/c mice or NOD/SCID mice and monitored every day for 3 weeks. JSI-124 (1 mg/kg day; Sigma, USA) or anti-IL-6R mAb (2.5 mg/kg week; Bio X Cell, USA) were administrated intraperitoneally into 4T1^WT^-bearing BALB/c mice for 13 days. Saline solution-treated mice were used as negative controls. Besides, CD11b^+^Gr-1^−^ MDSCs (2 × 10^6^/twice/week) isolated from 4T1^WT^ tumors were transferred to 4T1^IL-6low^-bearing mice. The tumor volume was calculated using the formula *V* = (π × *a* × *b*^2^)/6, where *a* is the length and *b* is the width of the tumor. The number of metastatic nodules in the lungs was calculated as previously described (8). The experiment was approved by the Ethics Committee for Animal Experiments at the Tianjin Medical University Cancer Hospital and Institute and was performed in accordance with the Guide for the Care and Use of Laboratory Animals.

### Isolation and Differentiation of Primary MDSCs *In Vivo*

Primary MDSCs in tumors and spleens were isolated from the single cell suspension of tumor tissues and spleens *via* magnetic bead enrichment as described previously (12). Briefly, both tumor tissues and spleens were dissociated into single cell suspensions (24). After erythrocytolysis, CD11b^+^Gr-1^+^ MDSCs were isolated using beads conjugated with biotin anti-mouse Gr-1 and anti-biotin microbeads (Miltenyi Biotec, Germany), and CD11b^+^Gr-1^−^ MDSCs were isolated using anti-mouse CD11b microbeads after CD11b^+^Gr-1^+^ MDSCs were removed. CD11b^+^Gr-1^−^F4/80^−^MHCII^−^ MDSCs were separated using the BD FACSAria™ II cell sorter (BD Biosciences, San Jose, CA, USA). The viability and purity of the recovered cells were determined using trypan blue staining assay and flow cytometry.

CD11b^+^Gr-1^−^ MDSCs isolated from tumors were labeled with CSFE (0.5 µM, Invitrogen, USA) for 20 min and transferred back to female BALB/c mice *via* tail vein. And 96 h later, spleen single cell suspensions were prepared, and the proportions of CSFE-labeled cells in CD11b^+^Gr-1^+^ subset were analyzed using flow cytometry.

### Proliferation and Immunosuppressive Capacity of Primary MDSCs

4T1^WT^-, 4T1^NC^-, and 4T1^IL-6low^-bearing mice were generated as previously reported, and BrdU (50 mg/kg) was injected by tail vein 3 weeks after tumor transplantation. And 72 h later, the percentage of BrdU-labeled CD3^+^ T cells, CD11b^+^Gr-1^+^ MDSCs, and CD11b^+^Gr-1^−^ MDSCs in tumors and spleens was analyzed using flow cytometry.

T cells, isolated from normal BALB/c mice using the Pan T Cell Isolation Kit (Miltenyi Biotec), were cocultured with either spleen- or tumor-derived MDSCs from tumor-bearing mice at a 1:3 ratio in RPMI-1640 medium supplemented with 10% FBS in 24-well plates. Anti-CD3/CD28 beads (20 μL/10^6^ cells; Gibco) were utilized to stimulate T cells *in vitro*. The proliferation and apoptosis of the T cells were examined using BrdU and Annexin V staining assay as described previously (7). Finally, the supernatants were collected to detect multiple cytokine levels using ELISA.

### Induction and Differentiation of MDSCs *In Vitro*

Bone marrow (BM) cells were flushed from mice femurs using PBS. Erythrocytolyzed cells were added to multi-well plates and cocultured with 4T1^NC^ or 4T1^IL-6low^ breast cancer cells at 1:1 ratio or fresh supernatant of 4T1^NC^ or 4T1^IL-6low^ or recombinant IL-6 (rIL-6, 50 ng/mL; PeproTech, USA) to induce MDSCs (iMDSCs). IL-6R Ab (10 µg/mL) was utilized to block IL-6 signaling pathway. Seventy-two hours later, the percentage of MDSCs was detected by flow cytometry. Besides, CD11b^+^Gr-1^−^ iMDSCs and BM cells stimulated by IL-6 (40 ng/mL) for 15 min were collected at different time point. Besides, MDSCs isolated from tumors were cultured in complete RPMI-1640 medium supplemented with 10% FBS and incubated in 5% CO_2_ at 37°C. GM-CSF (10 ng/mL; PeproTech, USA) was used to induce differentiation in MDSCs *in vitro*. Seventy-two hours later, the cells were collected to detect the expression of differentiation-related membrane molecules using flow cytometry and RT-PCR.

### Flow Cytometry Analysis

The single cell suspension obtained from the spleens and tumors was incubated with PerCP-conjugated anti-mouse CD45, FITC-conjugated anti-mouse CD11b, and PE-conjugated anti-mouse Gr-1 (RB6-8C5) antibodies to detect infiltrated MDSCs. Isolated CD11b^+^Gr-1^−^ MDSCs were labeled with PE/CY7-conjugated anti-mouse F4/80 and PE-conjugated anti-mouse MHCII antibodies (BioLegend, CA, USA). The MDSCs were cultured *in vitro* and incubated with PerCP-conjugated anti-mouse CD45, FITC-conjugated anti-mouse CD11b, PE-conjugated anti-mouse Gr-1 (RB6-8C5), PE/CY7-conjugated anti-mouse F4/80, PE-conjugated anti-mouse MHCII, and APC-conjugated anti-mouse Ly6G (1A8) (BioLegend) to detect the MDSCs and mature myeloid cells. To confirm the efficacy of JSI-124 and IL-6R blocking, intracellular staining assay was conducted using eBioscience™ Foxp3/Transcription Factor Staining Buffer Set (eBioscience, San Jose, CA, USA) to detect the changes of STAT and SOCS protein in MDSCs of tumors. MDSCs were labeled with p-STAT3, p-STAT1, SOCS3, or SOCS1 McAbs (CST, 1:200) and Goat anti-Rabbit PE/CY7-IgG (Bioss, Beijing, China, 1:100). The samples were then analyzed using the BD FACSCanto™ II flow cytometer (BD Biosciences, San Jose, CA, USA).

### Immunohistochemistry

Tumors harvested from the 4T1^WT^, 4T1^NC^, and 4T1^IL-6low^ mice models were fixed in 10% formaldehyde and embedded in paraffin. Tissue sections (4 µm) were deparaffinized in xylene and rehydrated through graded concentrations of ethanol. Antigens were retrieved and endogenous peroxidase activities were quenched as described previously (25). The tumor tissues were incubated overnight at 4°C with rabbit anti-mouse CD11b (1:500), Gr-1 (1:200), and CD3 (1:100) primary antibodies (Abcam, Cambridge, UK). A biotinylated secondary antibody, goat anti-mouse IgG (Santa Cruz Biotechnology, USA), was labeled with streptavidin–horseradish peroxidase (HRP) using a DAB staining kit (Maixin Biotechnology, China). Five high-power fields (400× magnification) for each tissue section were selected randomly for histological evaluation.

### Enzyme-Linked Immunosorbent Assay

Cells (4 × 10^5^) were seeded into 6-well plates, and the culture supernatants of 4T1^WT^, 4T1^NC^, and 4T1^IL-6low^ cells were collected after 48 h to determine the IL-6 levels using the mouse IL-6 ELISA kit (Dakewe Biotech Co., Ltd., Shenzhen, China). The supernatants of cocultured MDSCs and T cells were also collected, and the levels of IL-10, TGF-β, and IFN-γ were determined using the ELISA kit (Dakewe Biotech Co., Ltd.).

### Cell Counting Kit-8 (CCK-8) Assay

Cells (8 × 10^3^) were seeded into 96-well plates. CCK-8 solution (10 µL; Dojindo Molecular Technologies, Inc., Rockville, MD, USA) was added to each well for different time periods (2, 24, 72, and 96 h) and incubated for 2 h at 37°C. The cell viability was represented by the absorbance measured at 450 nm.

### Clonogenic Assay

Cells (400 cells/well) were seeded into 6-well plates and cultured in RPMI-1640 medium containing 10% FBS. After 10 days, the plates were washed with PBS. Colonies were fixed in 4% paraformaldehyde solution and stained with 1% crystal violet.

### Scratch Wound Healing Assay

Cells were cultured to confluency in serum-free medium for 12 h and then scratched with a sterile pipette tip. The cells were subsequently washed with PBS and then incubated with serum-free RPMI-1640 medium at 37°C. The wound areas were photographed and analyzed using IPP 6.0 at a 4× magnification. The migration rate was measured photogrammetrically at 24 and 48 h.

### Invasion Assay

Cells (1 × 10^4^) were suspended in 200 µL of serum-free RPMI-1640 medium and seeded into the top wells of the apparatus, which were coated with a 50 mg/L matrigel (1:7 diluted solution; BD Biosciences) and air dried at 37°C. The bottom well contained 500 µL of RPMI-1640 medium containing 10% FBS. After 48 h at 37°C and 5% CO_2_, the filtered cells were fixed in 4% paraformaldehyde solution and stained with 1% crystal violet. The stained cells from five selected views were observed under a light microscope at a 200× magnification.

### Quantitative Real-Time RT-PCR

Total cellular RNA was extracted using the Trizol-trichloromethane method, and RNA quantity was determined spectrophotometrically. Synthesis of cDNA was performed using the TaKaRa PrimeScript^®^ RT reagent Kit (TaKaRa Bio, Japan). Quantitative real-time PCR was performed using the SYBR Premix Ex Taq TM system (TaKaRa Bio) and primers shown in Table 1. The relative mRNA levels were calculated based on the threshold cycle (Ct) values normalized to the Ct value of β-actin using the following formula: 2^−ΔCt^, where ΔCt = Ct_target gene_ − Ct_β-actin_. All tests were performed in triplicate.

**Table 1:** The RT-PCR primers of interested genes.

Genes	Primer sequences	
IL-6	Forward Reverse	CGGAGAGGAGACTTCACAGAG ATTTCCACGATTTCCCAGAG
MPO	Forward Reverse	AGCTGACCAAGGACCAGGAG GCAGTTGAGGCCAGTGAAGA
TNF-α	Forward Reverse	ACTGGCAGAAGAGGCACTCC GCCACAAGCAGGAATGAGAA
SOCS1	Forward Reverse	CAACGGAACTGCTTCTTCG AAGGCAGTCGAAGGTCTCG
SOCS2	Forward Reverse	TGTGCAAGGATAAACGGACA AATGGCGAGTCGACAGAAAT
SOCS3	Forward Reverse	GGAGAGCGGATTCTACTGGA TGACGCTCAACGTGAAGAAG
β-Actin	Forward Reverse	GTGCTATGTTGCTCTAGACTTCG ATGCCACAGGATTCCATACC

### Western Blot Analysis

Cell lysates were resolved using SDS-PAGE and transferred to polyvinylidene difluoride membranes. The membranes were blocked with 5% BSA and then incubated overnight at 4°C with anti-SOCS1 (1:1,000; CST), anti-SOCS2 (1:1,000; CST), anti-SOCS3 (1:1,000; CST), anti-STAT3 (1:1,000; CST), anti-p-STAT3 (1:1,000; CST), anti-STAT1 (1:1,000; CST), anti-p-STAT1 (1:1,000; CST), JAK1 (1:1,000; CST), phosphorylated JAK1 (p-JAK1) (1:1,000; CST), JAK2 (1:1,000; CST), p-JAK2 (1:1,000; CST), TYK2 (1:1,000; CST), CD126 (1:1,000; Abcam, Cambridge, UK), gp130 (1:1,000; CST), p-P38 (1:1,000; CST), P38 (1:1,000; CST), p-JNK (1:1,000; CST), JNK (1:1,000; CST), ERK (1:1,000; CST), p-ERK (1:1,000; CST), or anti-β-actin (1:4,000; Santa Cruz Biotechnology) antibodies. Next, the membranes were incubated with HRP-conjugated anti-mouse or anti-rabbit IgG secondary antibodies (Zsbio, China) for 1 h. A SuperSignal West Pico chemiluminescent substrate kit (Pierce Biotechnology, Rockford, IL, USA) was used to visualize the protein bands. The relative densities of the bands were measured using the Quantity One software and normalized to the β-actin, STAT1, STAT3, JAK1, JAK2, TYK2, P38, JNK, and ERK bands. The validity of JAK/STAT and SOCS antibodies was verified according to the instruction manuals, and the details are presented in Supplemental data 3 in Supplementary Material.

### Statistical Analysis

Statistical analyses were performed using SPSS 20.0 and GraphPad Prism 5.0 softwares. The differences in measurement data were presented as mean ± SD. One-way analysis of variance and least significant difference tests were used to compare the quantitative data. *P* values for each analysis are reported in the figure legends. The level of statistical significance was set at *P* < 0.05.

## Results

### IL-6 Knockdown Repressed the Growth and Metastasis of 4T1 Tumors *In Vivo*

**Figure 1:**
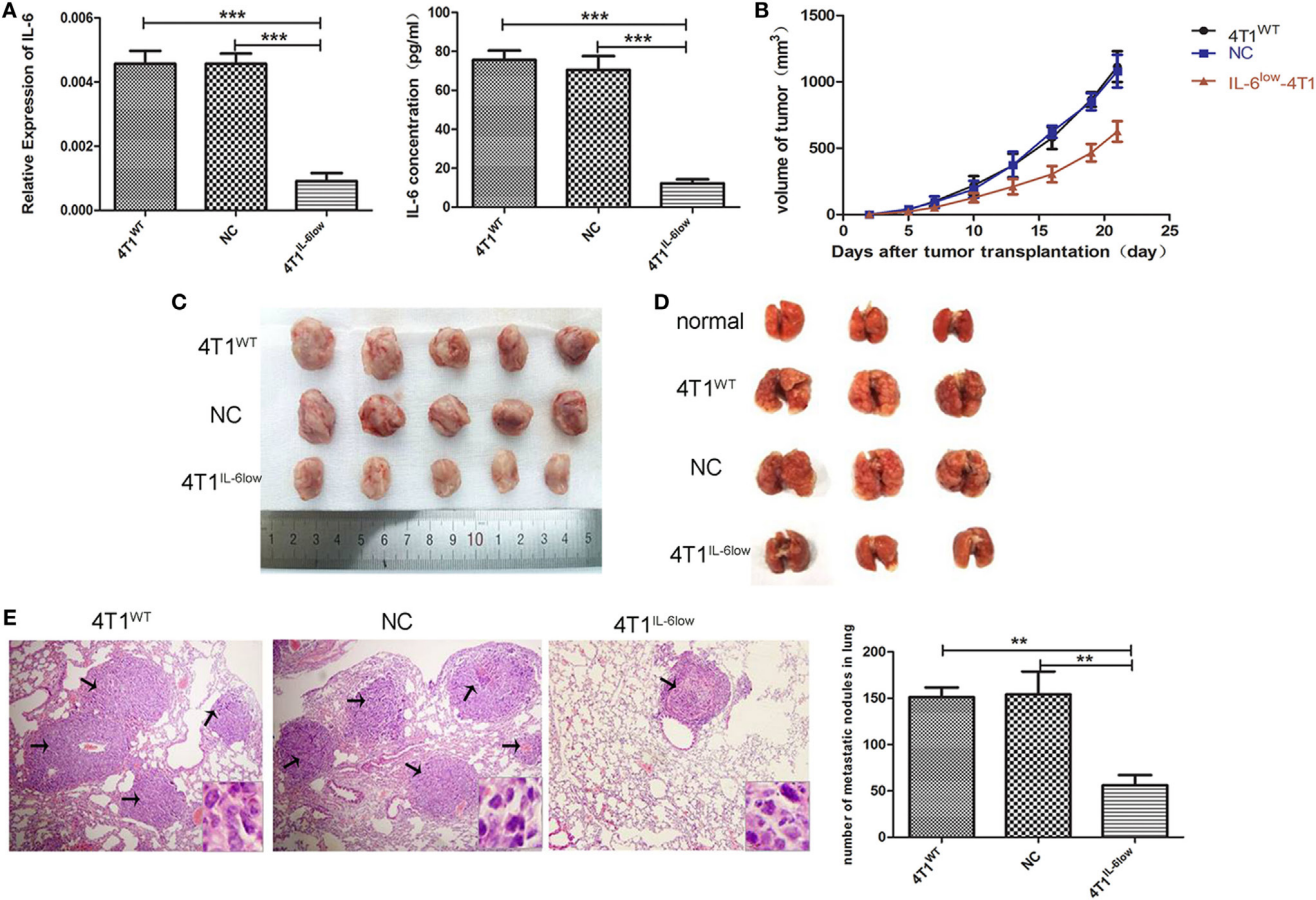
Decreased tumor-derived interleukin-6 (IL-6) significantly repressed 4T1 mammary tumor growth and metastasis *in vivo*. **(A)** Silencing efficiency of the IL-6-short hairpin RNA lentiviral vector was confirmed by RT-PCR and enzyme-linked immunosorbent assay. Cells (4T1^WT^, 4T1^NC^, and 4T1^IL-6low^) were injected into the mammary fat pads of BALB/c mice. **(B)** Tumor sizes were monitored and calculated using the following equation: *V* = ((π × *a* × *b*^2^)/6), where *a* is the length and *b* is the width of the tumor. Mice were sacrificed on day 21, and tumors **(C)** and lungs **(D)** were separated. **(E)** Pulmonary metastatic nodules were counted based on H&E-stained slides (*P < 0.05; **P < 0.01; and ***P < 0.001).

The murine mammary carcinoma cell line, 4T1, highly expressed IL-6 (13). Our preliminary study was consistent with previous reports and confirmed high expression levels of IL-6 at both the RNA and protein levels. In this study, the IL-6-knockdown in the 4T1 murine mammary carcinoma cell line (4T1^IL-6low^) was successfully established to investigate the effects of tumor-derived IL-6 on the growth and invasion of breast cancer cells by infecting lentiviral shIL-6 into mammary carcinoma 4T1 cells. Compared with the 4T1^WT^ and 4T1^NC^ cells, the 4T1^IL-6low^ cells expressed lower levels of IL-6 at both the RNA and protein levels (*P* = 0.0002, *P* = 0.0001; *P* < 0.001, *P* = 0.0002, Figure 1A). Furthermore, the 4T1^WT^, 4T1^NC^, and 4T1^IL-6low^ cells were implanted into BALB/c mice to generate mammary carcinoma-bearing models. We found that IL-6 knockdown strikingly inhibited tumor growth (*P* < 0.001, *P* < 0.001, Figure 1B). After the mice were sacrificed, the volume of primary tumors and number of lung metastatic nodules were calculated. The average volume of the 4T1^IL-6low^ tumors was lower than that of both 4T1^WT^ and 4T1^NC^ cells (585.2 ± 78.6 vs. 1,080.6 ± 115.7 vs. 1,122.6 ± 132.9 mm^3^, *P* = 0.0036, *P* = 0.0038, Figure 1C). Furthermore, the number of metastatic nodules in the lungs was also dramatically reduced in 4T1^IL-6low^ group than both the 4T1^WT^ and 4T1^NC^ groups (56.0 ± 6.4 vs. 151.0 ± 6.1 vs. 154.0 ± 14.3, *P* = 0.0004, *P* = 0.0033, Figures 1D,E). Furthermore, the 4T1^WT^, 4T1^NC^, and 4T1^IL-6low^ cells were implanted into NOD/SCID mice separately, and the results showed that IL-6 knockdown inhibited tumor growth to some extent in T- and B-cell immunity-deficient mice (*P* = 0.001). But less tumor suppression in NOD/SCID mice was observed compared with that in immunity competent mice which implied that except T and B cells, other immune cells, such as NK cells, may participate in 4T1 immune evasion (Supplemental data 4 in Supplementary Material). Therefore, these results implied that tumor-derived IL-6 significantly promoted the growth and metastasis of 4T1 mammary carcinoma *in vivo*.

### IL-6 Knockdown Did Not Affect the Growth and Invasion of 4T1 Cells *In Vitro*

**Figure 2:**
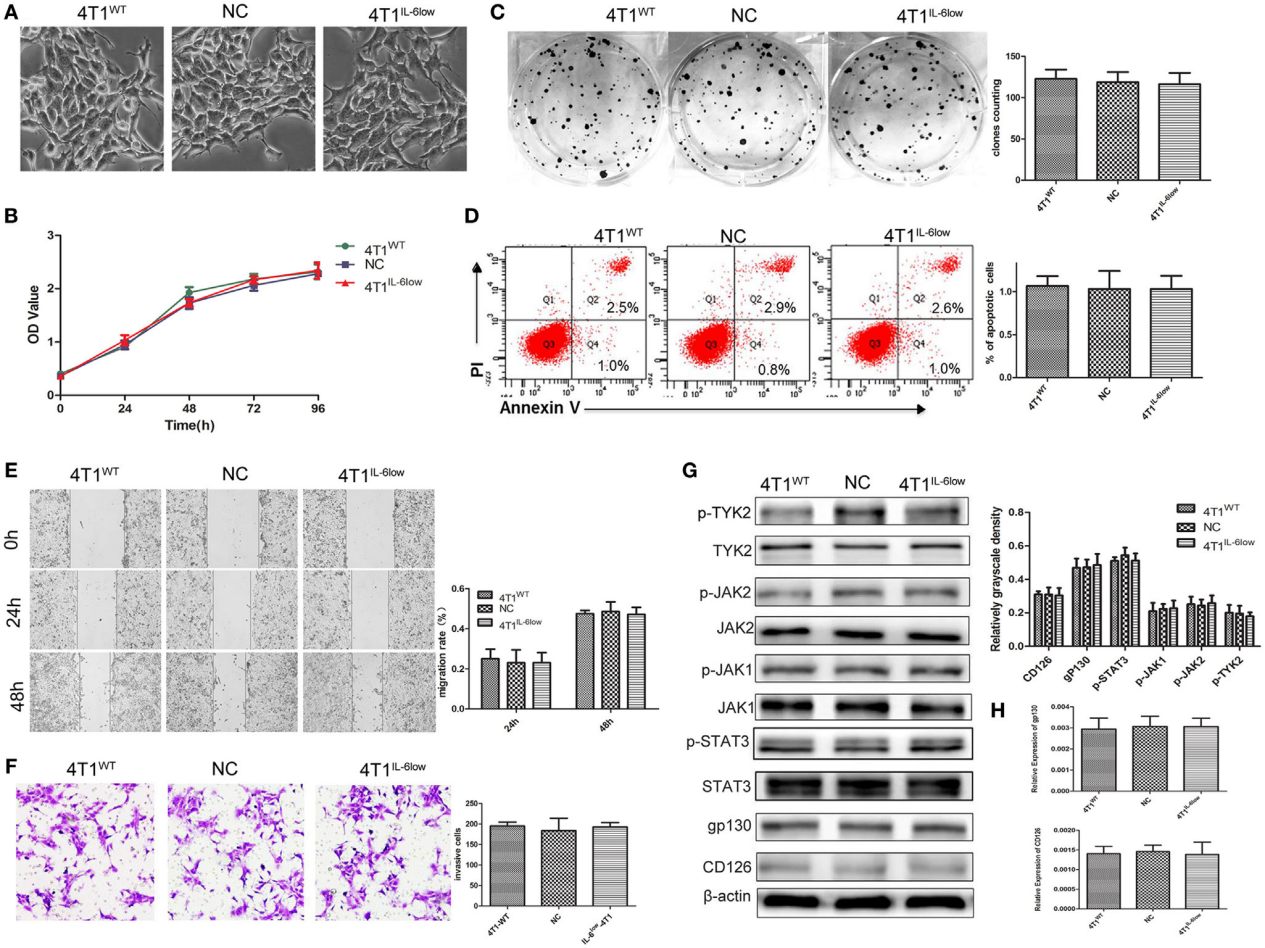
Effects of interleukin-6 (IL-6) knockdown in 4T1 cells *in vitro*. **(A)** Morphology of 4T1^WT^, 4T1^NC^, and 4T1^IL-6low^ cells. **(B)** Cell growth was monitored by Cell Counting Kit-8 assay. **(C)** Clonogenic survival analysis of infected 4T1 cells. **(D)** The quantification of the infected 4T1 cell apoptosis was detected using the Annexin V-FITC apoptosis detection kit. Apoptotic cells were recognized by Annexin V^+^PI^−^. **(E)** Wound healing assay was used to study the migratory ability of infected 4T1 cells (original magnification 40×). **(F)** The infiltrative and metastatic capability of infected 4T1 cells was evaluated by invasion assay (original magnification 10×). The stained cells from five selected views were observed under a light microscope at 200× magnification. **(G)** The downstream signaling pathway of IL-6 in 4T1^WT^, 4T1^NC^, and 4T1^IL-6low^ cells was detected. The levels of phosphorylated JAK1 (p-JAK1), p-JAK2, p-TYK2, and p-STAT3 were compared using the density ratio of phosphorylated protein to total protein. The levels of the CD126 and gp130 protein were compared using the density ratio of the indicated protein to β-actin. **(H)** The mRNA levels of gp-130 and CD126 in 4T1^WT^, 4T1^NC^ AND 4T1^IL-6low^ were determined by RT-PCR.

To determine whether IL-6 promoted tumor growth and metastasis by directly affecting the biological behavior of 4T1 cells, we examined the effects of IL-6 knockdown on the proliferation, apoptosis, migration, and invasion of 4T1 cells *in vitro*. These cells normally exhibited spindle-like features, and no morphological changes were observed after IL-6 knockdown (Figure 2A). We compared the proliferation of 4T1^WT^, 4T1^NC^, and 4T1^IL-6low^ cells using the CCK-8 staining assay and found no difference in their proliferation rates *in vitro* (*P* = 0.773, *P* = 0.461, Figure 2B). Furthermore, no disparity in colony numbers was observed between these three groups in the colony formation assay, which indicated that there was no detectable disparity in the colony formation capacity of 4T1 cells after IL-6 knockdown (*P* = 0.5686, *P* = 0.8369, Figure 2C). We also found that IL-6 knockdown had no effect on cell apoptosis in the Annexin V assay (*P* = 0.7780, *P* = 1.00, Figure 2D). Furthermore, cell migration and invasion of the 4T1^WT^, 4T1^NC^, and 4T1^IL-6low^ cells were compared using the wound healing and transwell assays, which demonstrated a similar migration pattern (*P* = 0.7953, *P* = 0.6537, Figure 2E) and invasive capacity (*P* = 0.8892, *P* = 0.7076, Figure 2F). These results implied that IL-6 knockdown did not affect the growth and invasion of 4T1 cells *in vitro*.

We also detected the IL-6 signaling pathway in 4T1^WT^, 4T1^NC^, and 4T1^IL-6low^ cells. And the results showed that JAK1, JAK2, TYK2, and STAT3 phosphorylation was observed, and no significant disparity was found among three groups (Figure 2G). Besides, we detected CD126 and gp130 in three cells. And the results showed that there was no significant difference in CD126 and gp130 expression both at mRNA and protein level (Figures 2G,H). Furthermore, IL-6 signaling pathway was further blocked using IL-6R antibody (10 µg/mL) in 4T1^IL-6low^ cells. The results showed that IL-6R Ab inhibited 4T1^IL-6low^ cells growth (*P* < 0.001) and colony formation (*P* = 0.0025) but had no effect on apoptosis (*P* = 0.9106). Besides, cell migration (*P* = 0.0345, *P* = 0.0024) and invasion of the 4T1^IL-6low^ cells (*P* = 0.0084) were significantly suppressed by IL-6R Ab (Supplemental data 2 in Supplementary Material). Above results implied that merely reducing the level of IL-6 did not affect the downstream JAK/STAT signaling pathway, but fully blocking the interaction between IL-6 and IL-6R was sufficient to inhibit the growth and invasion of 4T1 cells *in vitro*.

### IL-6 Promoted the Accumulation of CD11b^+^Gr-1^−^F4/80^−^MHC-II^−^ e-MDSCs in Tumors

**Figure 3:**
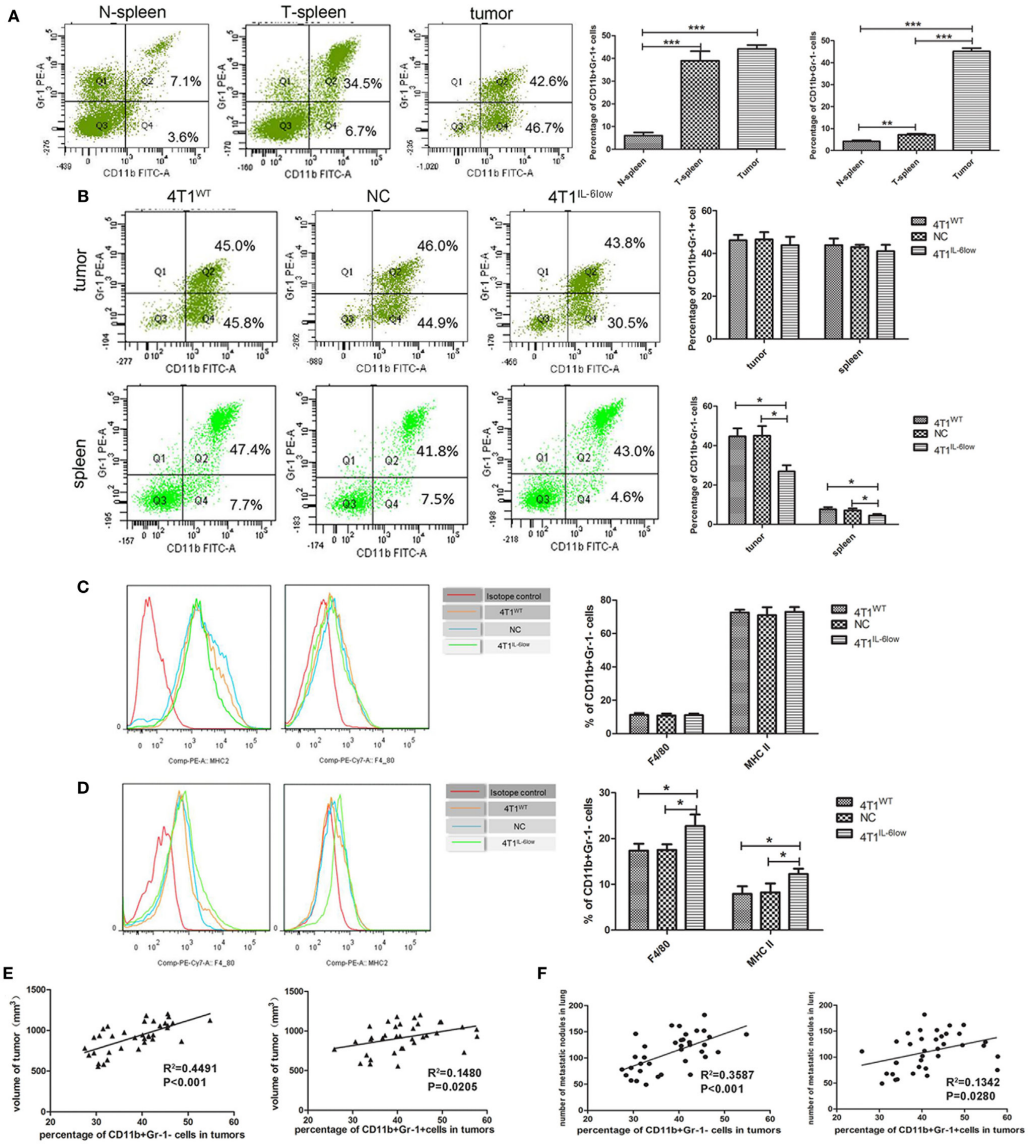

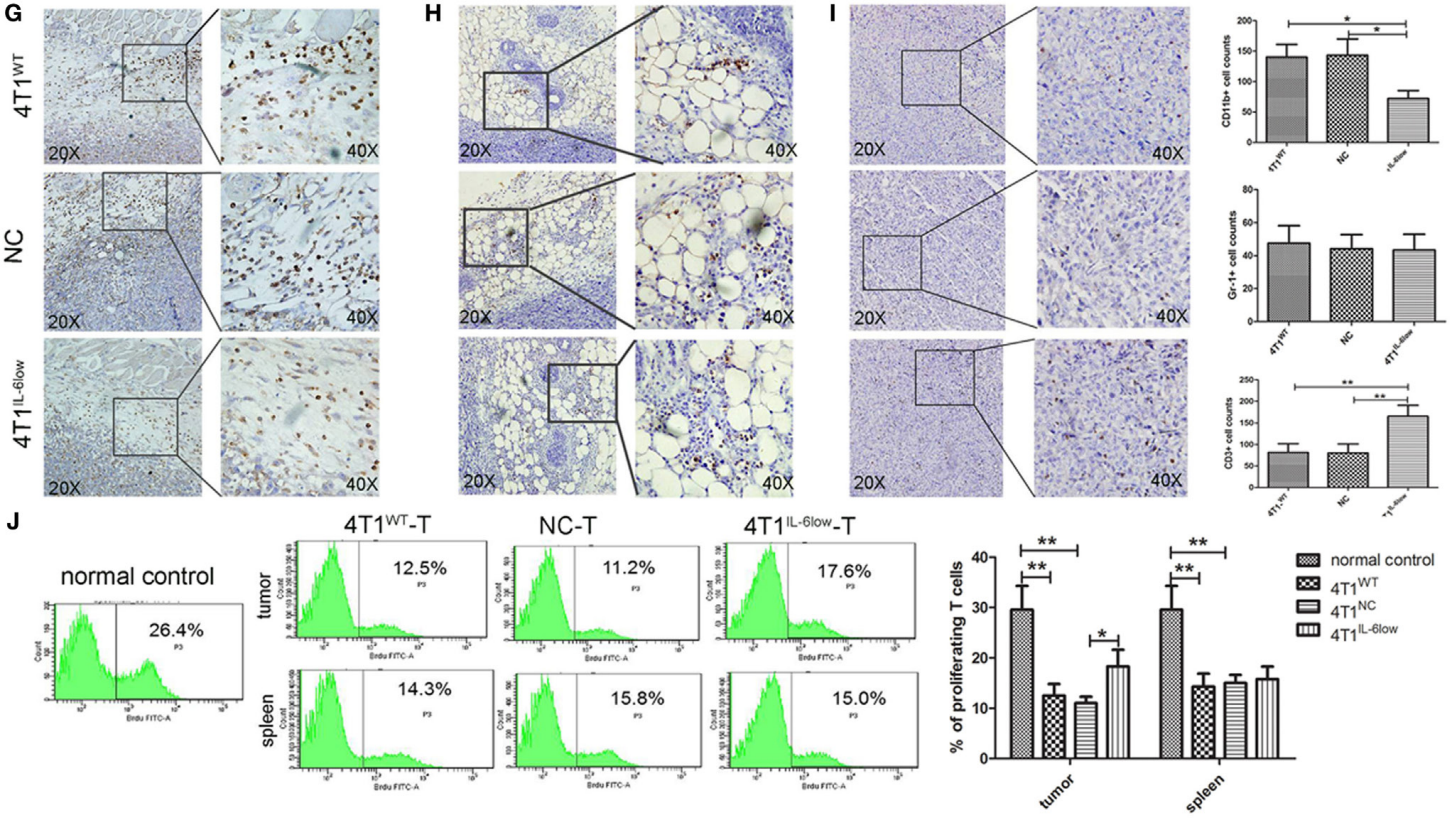
Tumor-derived interleukin-6 (IL-6) promotes the accumulation of CD11b^+^Gr-1^−^F4/80^−^MHCII^−^ early-stage MDSCs (e-MDSCs) in tumors. Infiltrated CD45^+^ cells were gated. **(A)** The percentage of myeloid-derived suppressor cells (MDSCs) in normal spleen, tumors, and spleens resulting from 4T1 mammary cancer cells detected by flow cytometry. **(B)** The percentage of the indicated cell subsets in tumors and spleens from mice bearing different IL-6-expressing tumors was determined by flow cytometry. CD11b^+^Gr-1^−^ e-MDSCs from spleens and tumors were isolated, and the percentage of CD11b^+^Gr-1^−^F4/80^+^MHCII^−^ cells and CD11b^+^Gr-1^−^F4/80^−^MHCII^+^ cells in spleens **(C)** and tumors **(D)** was determined using flow cytometry. **(E)** The correlation analysis between tumor volumes and the percentage of tumor-infiltrating CD11b^+^Gr-1^−^F4/80^−^MHCII^−^ e-MDSCs or CD11b^+^Gr-1^+^ MDSCs (*R*^2^ = 0.4491 vs. *R*^2^ = 0.1482, *P* < 0.001, *P* < 0.05). **(F)** Correlation analysis between lung metastatic nodules and the percentage of tumor-infiltrating CD11b^+^Gr-1^−^F4/80^−^MHCII^−^ e-MDSCs or CD11b^+^Gr-1^+^ MDSCs (*R*^2^ = 0.3587 vs. *R*^2^ = 0.1342, *P* = 0.0001, *P* < 0.05). Infiltration of CD11b^+^ cells **(G)**, Gr-1^+^ cells **(H)**, and CD3^+^ T cells **(I)** in tumors expressing different amounts of IL-6 was determined by immunohistochemistry. Five high-power fields (400× magnification) for each tissue section were selected randomly for histology evaluation. **(J)** BrdU (50 mg/kg) was injected by tail vein separately to mice bearing 4T1^WT^, 4T1^NC^, or 4T1^IL-6low^ tumors, and proliferative T cells labeled with BrdU in tumors and spleens were detected. T cells in spleen of normal mice were used as control (*P < 0.05; **P < 0.01; and ***P < 0.0001).

In our previous study, we demonstrated that tumor-derived IL-6 positively correlated with MDSC infiltration *in situ* in human primary breast cancer tissues, which correlated with more aggressive tumor phenotypes and worse clinical outcomes (9). Therefore, we compared the relationship between IL-6 levels and MDSCs infiltration in primary 4T1 tumors, along with the size and number of tumors and metastatic lung nodules. Flow cytometry analysis revealed that CD11b^+^Gr-1^+^ MDSCs were higher in the tumor tissues and spleens of tumor-bearing mice when compared with the same in normal controls (44.13 ± 1.00 vs. 39.03 ± 2.38 vs. 6.03 ± 0.79%, *P* = 0.0002, *P* < 0.001). However, a significant increase in CD11b^+^Gr-1^−^ cells was exclusively observed in the tumor tissues and spleens of tumor-bearing mice when compared with the same in normal controls (45.13 ± 0.85 vs. 7.20 ± 0.30 vs. 4.17 ± 0.30%, *P* < 0.0001, *P* = 0.0019) (Figure 3A).

After IL-6 knockdown, CD11b^+^Gr-1^−^ MDSCs decreased significantly in both the tumor tissues (26.90 ± 4.81 vs. 45.03 ± 4.84 vs. 44.67 ± 3.34%, *P* = 0.0039, *P* = 0.0057) and spleens (4.48 ± 0.47 vs. 7.53 ± 0.55 vs. 7.67 ± 0.61%, *P* = 0.0142, *P* = 0.0208) of the 4T1^IL-6low^ group when compared with that of the 4T1^WT^ and 4T1^NC^ groups. Meanwhile, no significant difference in the percentage of CD11b^+^Gr-1^+^ MDSCs was observed in either the tumor tissues (43.90 ± 2.22 vs. 46.15 ± 1.46 vs. 46.50 ± 1.967%, *P* = 0.4452, *P* = 0.4306) or spleens (41.03 ± 1.72 vs. 43.90 ± 1.76 vs. 42.93 ± 0.65%, *P* = 0.3093, *P* = 0.3603) of the three groups (Figure 3B). Using Gr-1^−^F4/80^+^ as the characteristic phenotype of mature macrophages and Gr-1^−^MHCII^+^ as the characteristic phenotype of dendritic cells (DCs) as in previous studies (1, 26, 27), we compared the expression of F4/80 and MHCII in CD11b^+^Gr-1^−^ cells. We found that 70.73 ± 1.30% of the CD11b^+^Gr-1^−^ cells were F4/80^−^ and MHCII^−^ in tumors, which was the same as the e-MDSCs subset in mice, compared with only 15.10 ± 3.10% of CD11b^+^Gr-1^−^ cells being F4/80^−^ and MHCII^−^ in the spleens of tumor-bearing mice (Figures 3C,D). These results showed that CD11b^+^Gr-1^−^F4/80^−^MHC-II^−^ immature myeloid cells were enriched in tumors rather than in spleens. Furthermore, the percentage of Gr-1^−^F4/80^+^ cells (10.80 ± 1.20 vs. 11.00 ± 1.10 vs. 11.25 ± 1.15%, *P* = 0.8896, *P* = 0.9135) and Gr-1^−^MHC-II^+^ cells (73.97 ± 1.44 vs. 75.27 ± 2.95 vs. 72.57 ± 1.68%, *P* = 0.9181, *P* = 0.7433) in spleens displayed no significant difference among the 4T1^IL-6low^, 4T1^WT^, and 4T1^NC^ groups (Figure 3C). However, the percentage of Gr-1^−^F4/80^+^ (22.73 ± 1.45 vs. 17.37 ± 0.8570 vs. 17.50 ± 0.72%, *P* = 0.0335, *P* = 0.0321) and Gr-1^−^MHC-II^+^ (12.23 ± 0.68 vs. 7.90 ± 0.96 vs. 8.23 ± 1.14%, *P* = 0.0392, *P* = 0.0392) cells increased significantly in tumors in the 4T1^IL-6low^ group when compared with the 4T1^WT^ and 4T1^NC^ groups (Figure 3D). These results implied that tumor-derived IL-6 simulated the amplification and recruitment of immature CD11b^+^Gr-1^−^F4/80^−^MHC-II^−^ e-MDSCs instead of the conventional CD11b^+^Gr-1^+^ MDSCs exclusively in tumor tissues.

### CD11b^+^Gr-1^−^F4/80^−^MHC-II^−^ e-MDSCs Positively Correlated with Growth and Metastasis *In Vivo*

Next, the correlation among different MDSCs subsets, primary tumors sizes, and metastatic lung nodules was compared in the 4T1^WT^, 4T1^NC^, and 4T1^IL-6low^ groups. The percentage of CD11b^+^Gr-1^+^ MDSCs and CD11b^+^Gr-1^−^F4/80^−^MHC-II^−^ e-MDSCs significantly correlated with tumor volumes, i.e., the *in situ* sites that were infiltrated by higher numbers of CD11b^+^Gr-1^+^ MDSCs or CD11b^+^Gr-1^−^F4/80^−^MHC-II^−^ e-MDSCs showed larger tumor sizes (*R*^2^ = 0.1480 vs. *R*^2^ = 0.4491, *P* = 0.0205, *P* < 0.001, Figure 3E) and more metastatic lung nodules (*R*^2^ = 0.1342 vs. *R*^2^ = 0.3587 *P* = 0.0280, *P* = 0.0001, Figure 3F). However, CD11b^+^Gr-1^−^F4/80^−^MHC-II^−^ e-MDSCs displayed higher correlation with tumor volumes and lung metastases than CD11b^+^Gr-1^+^ MDSCs. In the IHC staining assay, CD11b^+^ MDSCs and Gr-1^+^ MDSCs were scattered in the stroma of the breast cancer tissues. Consistently, fewer CD11b^+^ MDSCs locally infiltrated the 4T1^IL-6low^ tumor tissues when compared with the 4T1^WT^ and 4T1^NC^ tumor tissues (72.20 ± 5.74 vs. 140.20 ± 9.33 vs. 143.20 ± 11.96, *P* = 0.0003, *P* = 0.0007, Figure 3G). However, no significant difference in the number of tumor-infiltrated Gr-1^+^ MDSCs was observed among the 4T1^WT^, 4T1^NC^, and 4T1^IL-6low^ tumor tissues (47.60 ± 4.729 vs. 44.20 ± 3.80 vs. 43.40 ± 4.27, *P* = 0.5284, *P* = 0.8922, Figure 3H). The above data implied that the CD11b^+^Gr-1^−^F4/80^−^MHC-II^−^ e-MDSCs were largely responsible for IL-6-stimulated tumor growth and metastasis.

Furthermore, fewer CD3^+^ T cells infiltrated the 4T1^WT^ and 4T1^NC^ tumor tissues when compared with the 4T1^IL-6low^ tumors (81.50 ± 10.11 vs. 80.25 ± 10.43 vs. 165.0 ± 12.91, *P* = 0.0022, *P* = 0.0022, Figure 3I). To determine the T cell function *in vivo*, BrdU (50 mg/kg) was injected by tail vein separately to mice bearing 4T1^WT^, 4T1^NC^, or 4T1^IL-6low^ tumors, and proliferative T cells labeled with BrdU in tumors and spleens were detected. T cells in spleen of normal mice were used as control. The results showed that T cell proliferation was suppressed dramatically in 4T1^WT^ and 4T1^NC^ tumors compared with normal controls (12.50 ± 1.33 vs. 11.03 ± 0.73 vs. 29.53 ± 2.74%, *P* = 0.0050, *P* = 0.0029). But the proliferation of T cells in 4T1^IL-6low^ tumors was recovered compared with that in 4T1^NC^ tumors (18.30 ± 1.91 vs. 11.03 ± 0.73, *P* = 0.0236), which implied that IL-6 reduction in tumor microenvironment can relieve immunosuppression on T cell proliferation and function. Meanwhile, the proliferation of T cells in spleens was also detected. And the results showed that the proliferation of T cells in 4T1^WT^ and 4T1^NC^ spleens was dramatically inhibited compared with normal controls (14.30 ± 1.51 vs. 15.00 ± 0.93 vs. 29.53 ± 2.74%, *P* = 0.0082, *P* = 0.0074, Figure 3J). But IL-6 knockdown has less effect on T cell proliferation of spleens compared with that in 4T1^NC^ spleens (15.80 ± 1.44 vs. 15.00 ± 0.93%, *P* = 0.6649, Figure 3J). Above data suggested that tumor-derived IL-6 mainly affected the immune state in tumors, rather than the systematic environment.

### CD11b^+^Gr-1^−^F4/80^−^MHC-II^−^ e-MDSCs Could Switch to CD11b^+^Gr-1^+^ MDSCs and Exerted Stronger T Cell Immunosuppression

**Figure 4:**
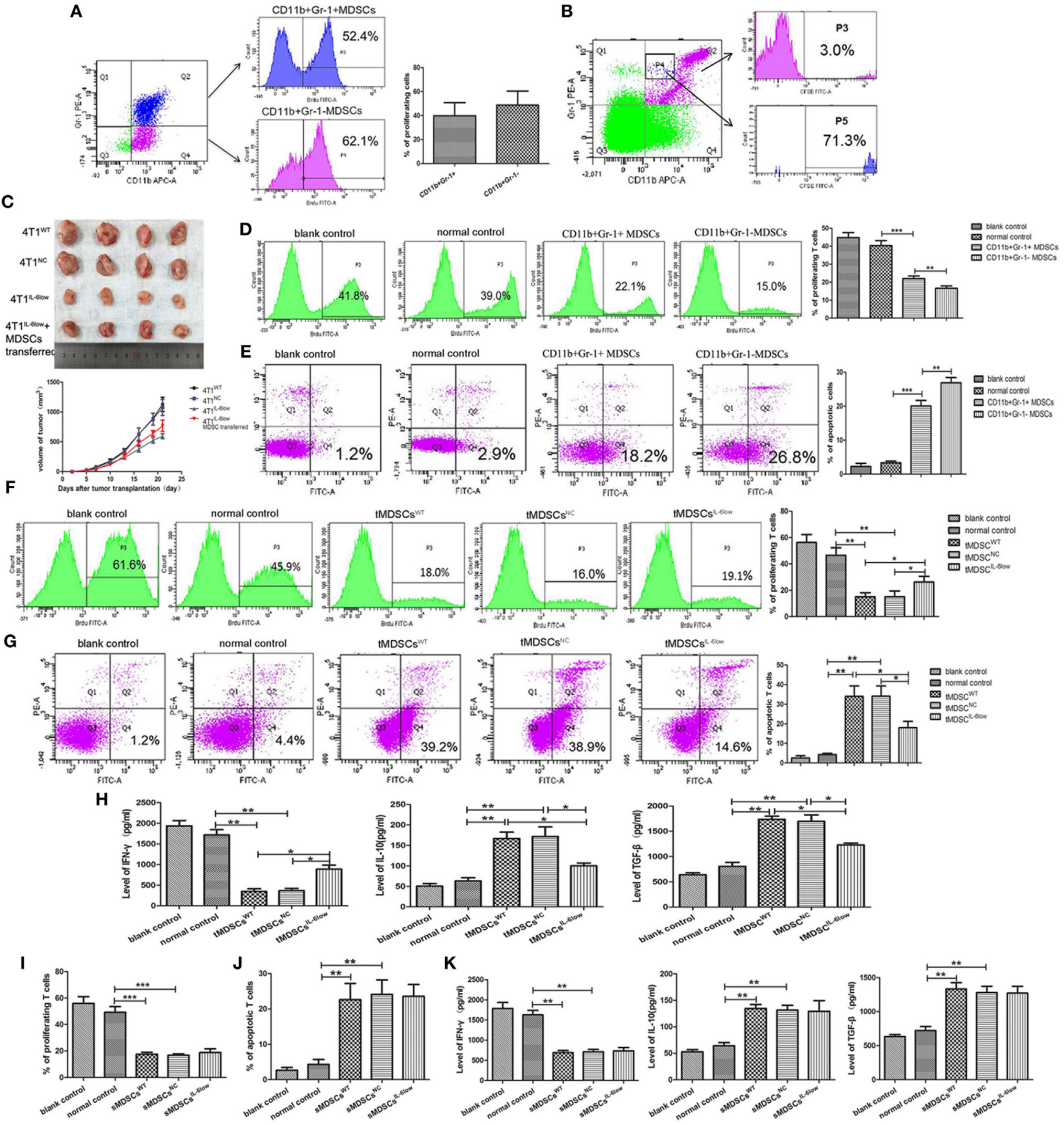
CD11b^+^Gr-1^−^F4/80^−^MHCII^−^ early-stage MDSCs (e-MDSCs) could switch to CD11b^+^Gr-1^+^ myeloid-derived suppressor cells (MDSCs) and exerted stronger T cell immunosuppression. **(A)** BrdU (50 mg/kg) was injected in 4T1^WT^-bearing mice by tail vein. Seventy-two hours later, CD11b^+^Gr-1^−^ MDSCs and CD11b^+^Gr-1^+^ MDSCs labeled with BrdU in tumors were detected by flow cytometry. **(B)** CD11b^+^Gr-1^−^ cells were isolated from 4T1^WT^ tumors and labeled with CSFE (0.5 µM) for 20 min *in vitro*. Then, the labeled cells were transferred back to normal BALB/c mice. After 96 h, CD11b^+^Gr-1^+^ cells in spleens were gated, and cells labeled with CSFE were detected. **(C)** CD11b^+^Gr-1^−^ MDSCs were separated from 4T1^WT^ tumors and transferred into mice bearing 4T1^IL-6low^ tumors (2 × 10^6^/twice/week), and the size of tumors was monitored. CD11b^+^Gr-1^+^ MDSC or CD11b^+^Gr-1^−^ e-MDSC was isolated from tumors and incubated with T cells *in vitro*. **(D)** The proliferation of T cells was detected by flow cytometry for Brdu^+^ cells. **(E)** Apoptotic T cells (Annexin V^+^PI^−^) were determined by flow cytometry. tMDSCs^WT^, tMDSCs^NC^, or tMDSCs^IL-6low^ were isolated from tumors and cocultured with T cells, respectively. **(F)** The proliferation of T cells was detected by flow cytometry for Brdu^+^ cells. **(G)** Apoptotic T cells (Annexin V^+^PI^−^) were determined by flow cytometry. **(H)** IFN-γ, IL-10, and TGF-β secreted by T cells were detected using enzyme-linked immunosorbent assay (ELISA). sMDSCs^WT^, sMDSCs^NC^, or sMDSCs^IL-6low^ were isolated from spleens and cocultured with T cells, respectively. **(I)** The proliferation of T cells was detected by flow cytometry for Brdu^+^ cells. **(J)** Apoptotic T cells (Annexin V^+^PI^−^) were determined by flow cytometry. **(K)** IFN-γ, IL-10, and TGF-β secreted by the T cells were detected using ELISA (**P < 0.01 and ***P < 0.001).

To determine the proliferation status of CD11b^+^Gr-1^−^ and CD11b^+^Gr-1^+^ MDSCs *in vivo*, BrdU (50 mg/kg) was injected by tail vein. Seventy-two hours later, MDSCs labeled with BrdU in tumors were detected by flow cytometry. The results showed that the percentage of BrdU-labeled CD11b^+^Gr-1^−^ e-MDSCs was higher than CD11b^+^Gr-1^+^ MDSCs (39.77 ± 6.33 vs. 48.70 ± 6.75, *P* = 0.3889), but no significance was observed (Figure 4A). Above results implied that CD11b^+^Gr-1^−^ MDSCs possess strong proliferative potential.

To determine if CD11b^+^Gr-1^−^ cells could switch to CD11b^+^Gr-1^+^ MDSCs *in vivo*, CD11b^+^Gr-1^−^ cells were isolated from tumors and labeled with CSFE (0.5 µM) for 20 min *in vitro*. Then, the labeled cells were transferred back to normal BALB/c mice. Seventy-two hours later, spleen single cell suspensions were prepared and the proportions of CSFE-labeled cells in CD11b^+^Gr-1^+^ subset were analyzed using flow cytometry. The results showed that 3.0% of CD11b^+^Gr-1^+^ cells was labeled with CSFE. Furthermore, CD11b^+^Gr-1^+^ cells transformed from CD11b^+^Gr-1^−^ cells were rich in gated P4, since 71.3% of cells in gated P4 was CSFE-labeled. All above data indicated that transferred exogenous CD11b^+^Gr-1^−^ cells were capable of inducing CD11b^+^ Gr-1^+^ cells *in vivo* (Figure 4B).

Furthermore, we determined whether e-MDSCs could promote tumor growth *in vivo*. 4T1^WT^, 4T1^NC^, and 4T1^IL-6low^ were transplanted in female BALB/c mice. Five days after transplantation, CD11b^+^Gr-1^−^ MDSCs were separated from 4T1^WT^ tumors and transferred into mice bearing 4T1^IL-6low^ tumors (2 × 10^6^/twice/week) by tail vein. The results showed that CD11b^+^Gr-1^−^ MDSCs transfer significantly promoted the growth of 4T1^IL-6low^ tumors (*P* = 0.008, Figure 4C). Above results showed that CD11b^+^Gr-1^−^ MDSCs transfer could reverse IL-6-knockwown mediated tumor growth suppression.

Considering that CD11b^+^Gr-1^−^F4/80^−^MHC-II^−^ e-MDSCs account for 70% of the CD11b^+^Gr-1^−^ MDSCs, CD11b^+^Gr-1^−^ MDSCs were isolated to represent the CD11b^+^Gr-1^−^F4/80^−^MHC-II^−^ e-MDSCs. CD11b^+^Gr-1^+^ MDSCs and CD11b^+^Gr-1^−^ e-MDSCs were isolated from 4T1^WT^ tumors and cocultured with normal spleen T cells. The CD11b^+^Gr-1^+^ MDSCs isolated from normal spleens were applied as normal controls. The proliferation and apoptosis of the T cells were examined separately. Tumor-derived CD11b^+^Gr-1^−^ e-MDSCs suppressed T cells more potently, which was demonstrated by the significant higher inhibition of the anti-CD3/CD28 McAb-stimulated T cell proliferation (15.10 ± 2.50 vs. 23.90 ± 1.48 vs. 46.50 ± 3.34% *P* = 0.0017, *P* = 0.0386, Figure 4D), and induced more apoptotic T cells (26.90 ± 0.87 vs. 20.03 ± 0.97 vs. 3.33 ± 0.30%, *P* < 0.001, *P* = 0.0063, Figure 4E) when compared with the CD11b^+^Gr-1^+^ MDSCs or normal controls. The aforementioned results implied that CD11b^+^Gr-1^−^ e-MDSCs exerted stronger T cell immunosuppressive abilities than conventional CD11b^+^Gr-1^+^ MDSCs.

### IL-6 Enhanced T Cell Immunosuppressive Capacity of e-MDSCs *In Vitro*

To study the IL-6-mediated regulation of the e-MDSC immunosuppressive capacity, CD11b^+^Gr-1^−^ e-MDSCs isolated from the 4T1^IL-6low^ tumors (tMDSC^IL-6low^), 4T1^WT^ tumors (tMDSC^WT^), and 4T1^NC^ tumors (tMDSC^NC^) were cocultured with normal spleen T cells to individually investigate proliferation, apoptosis, and cytokine production. The tMDSC^IL-6low^ showed dramatically alleviated suppression of T cell proliferation (26.23 ± 2.54 vs. 13.27 ± 0.91 vs. 12.23 ± 1.41%, *P* = 0.0222, *P* = 0.00352, Figure 4F) and attenuated stimulation of T cell apoptosis (17.97 ± 1.88 vs. 33.97 ± 3.09 vs. 34.07 ± 3.03%, *P* = 0.0115, *P* = 0.0107, Figure 4G) when compared with both tMDSC^NC^ and tMDSC^WT^. Furthermore, tMDSC^IL-6low^ induced more IFN-γ (888.90 ± 68.35 vs. 348.90 ± 37.81 vs. 367.10 ± 40.53 pg/mL, *P* = 0.0046, *P* = 0.0224), but lesser IL-10 (100.30 ± 4.83 vs. 167.0 ± 11.07 vs. 172.10 ± 16.51 pg/mL, *P* = 0.0313, *P* = 0.0200) and TGF-β (1,230 ± 25.18 vs. 1,739.0 ± 43.39 vs. 1,697.0 ± 91.25 pg/mL, *P* = 0.0096, *P* = 0.0388) secretion in T cells when compared with both tMDSC^NC^ and tMDSC^WT^ (Figure 4H).

CD11b^+^Gr-1^−^ e-MDSCs were also isolated from the spleens of 4T1^WT^ (sMDSC^WT^), 4T1^NC^ (sMDSC^NC^), and 4T1^IL-6low^ (sMDSC^IL-6low^) tumor-bearing mice and cocultured with normal spleen T cells to individually investigate proliferation, apoptosis, and cytokine production. No significant disparity in proliferation (17.57 ± 0.84 vs. 16.80 ± 0.61 vs. 18.80 ± 1.67%, *P* = 0.4812, *P* = 0.2924, Figure 4I) and apoptosis (22.63 ± 2.63 vs. 24.10 vs. 2.37 vs. 23.57 ± 1.94%, *P* = 0.7681, *P* = 0.9479, Figure 4J) was observed among the sMDSC^WT^, sMDSC^NC^, and sMDSC^IL-6low^ groups. Similarly, no significant changes in the secretion of IFN-γ (687.6 ± 32.97 vs. 708.8 ± 41.50 vs. 732.8 ± 57.31 pg/mL, *P* = 0.5079, *P* = 0.7662), IL-10 (134.6 ± 5.52 vs. 131.7 ± 6.370 vs. 129.3 ± 14.23 pg/mL, *P* = 0.7587, *P* = 0.8926), and TGF-β (1,333 ± 65.36 vs. 1,281 ± 63.93 vs. 1,272 ± 70.40 pg/mL, *P* = 0.5904, *P* = 0.9317) were determined in T cells stimulated by sMDSC^WT^, sMDSC^NC^, or sMDSC^IL-6low^ (Figure 4K). These results implied that tumor-derived IL-6 exclusively affected the immunosuppressive capacity of tumor CD11b^+^Gr-1^−^ e-MDSCs, which exerted stronger suppressive effects on T cell immunity than CD11b^+^Gr-1^+^ MDSCs.

### Reversible Myeloid Differentiation Blockage of e-MDSCs Is IL-6 Dependent

**Figure 5:**
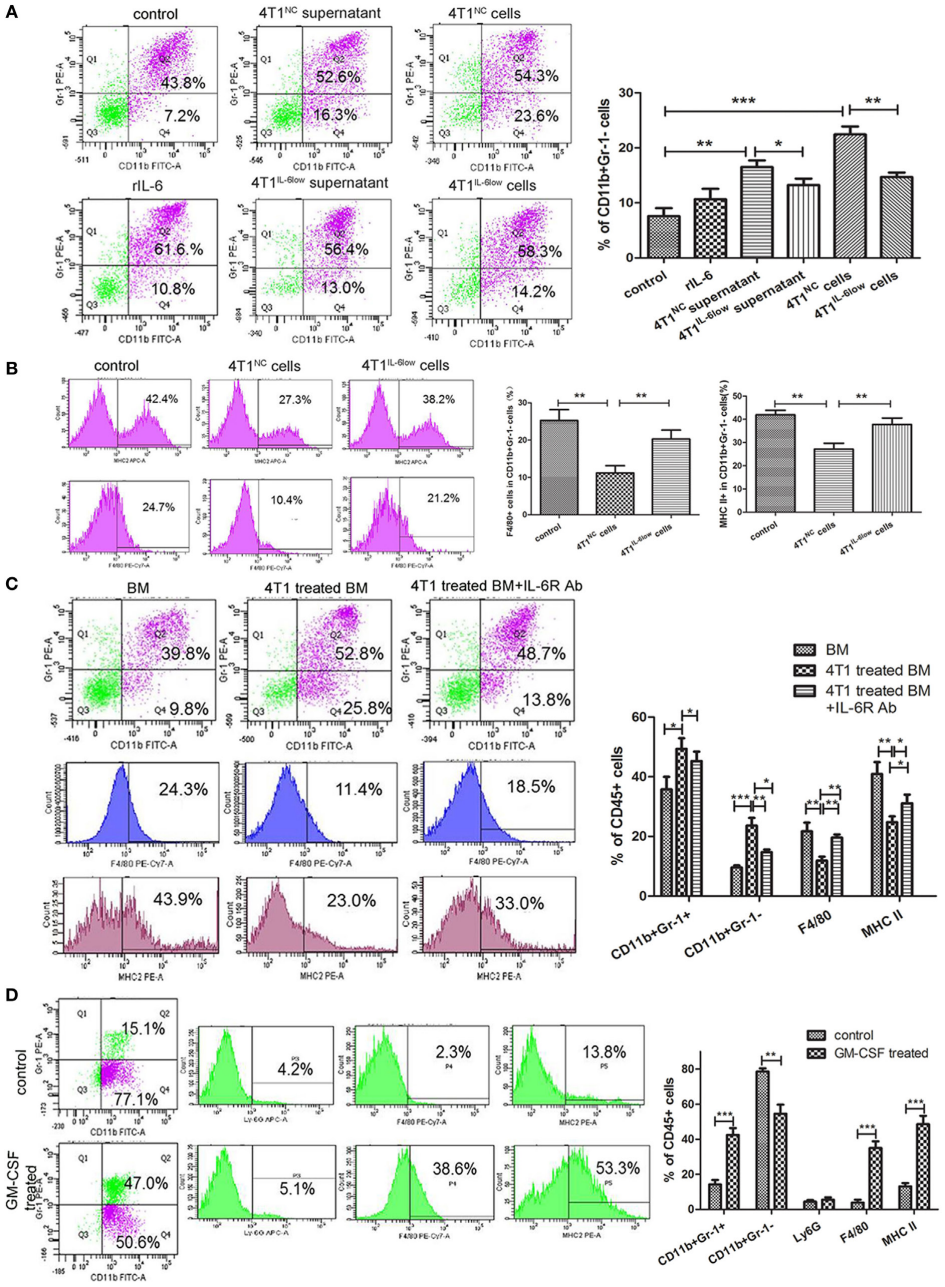
Reversible myeloid differentiation blockage of early-stage MDSCs (e-MDSCs) is interleukin-6 (IL-6) dependent. Bone marrow (BM) cells isolated from tumor-free BALB/c mice were cocultured with rIL-6, tumor cell supernatant, and tumor cells to induce e-MDSCs *in vitro*. And BM cells treated with GM-CSF (10 ng/mL) were regarded as normal control. **(A)** The percentage of CD11b^+^Gr-1^−^ was detected by flow cytometry. **(B)** The percentage of F4/80^+^ and MHCII^+^ cells in CD11b^+^Gr-1^−^ myeloid-derived suppressor cells (MDSCs) was detected using flow cytometry. BM cells were isolated from tumor-free mice and cultured with GM-CSF for 3 days with or without 4T1 cells. IL-6 receptor Ab was utilized to block IL-6 downstream signaling. **(C)** The percentage of CD11b^+^Gr-1^−^, CD11b^+^Gr-1^+^, F4/80^+^, and MHCII^+^ cells was detected using flow cytometry. **(D)** Primary CD11b^+^Gr-1^−^F4/80^−^MHCII^−^ cells were isolated using the BD FACSAria™II cell sorter and induced with GM-CSF. The percentage of CD11b^+^Gr-1^−^, CD11b^+^Gr-1^+^, Ly6G (1A8)^+^ cells, F4/80^+^ cells, and MHCII^+^ cells was detected by flow cytometry (*P < 0.05; **P < 0.01; and ***P < 0.001).

In this study, BM cells isolated from tumor-free BALB/c mice were cocultured with rIL-6, tumor cell supernatant and tumor cells to induce e-MDSCs *in vitro*. And BM cells treated with GM-CSF (10 ng/mL) were regarded as normal control. The results showed that rIL-6 solely had less effect on e-MDSCs development, since the percentage of CD11b^+^Gr-1^−^ MDSCs did not increase significantly compared with controls (10.67 ± 1.10 vs. 7.60 ± 0.83, *P* = 0.1002, Figure 5A). Both tumor supernatant and tumor cells could promote e-MDSC accumulation (16.50 ± 1.70 vs. 8.80 ± 1.29%, *P* = 0.0063; 22.43 ± 1.84 vs. 8.80 ± 1.29%, *P* = 0.0056, Figure 5A). Besides, e-MDSCs decreased significantly in 4T1^IL-6low^ cells group compared with 4T1^NC^ cells group (22.43 ± 0.84 vs. 14.70 ± 0.47%, *P* = 0.0013, Figure 5A). We concluded that IL-6-related tumor-derived soluble factors, but not IL-6 directly, affected the development of e-MDSCs. Furthermore, the percentage of MHC II positive or F4/80 positive cells in e-MDSCs was determined. The results showed that MHC-II^+^ cells (27.13 ± 1.48 vs. 41.93 ± 1.12%, *P* = 0.0013) and F4/80^+^ cells (11.20 ± 1.11 vs. 25.27 ± 1.67%, *P* = 0.0022) dramatically reduced in e-MDSCs induced by 4T1 cells compared with normal controls. But MHC-II^+^ cells (37.77 ± 1.60 vs. 27.13 ± 1.48%, *P* = 0.0081) and F4/80^+^ cells (20.30 ± 1.38 vs. 11.20 ± 1.11%, *P* = 0.0068) significantly increased in e-MDSCs induced by 4T1^IL-6low^ cells compared with 4T1^NC^ cells (Figure 5B). Above data suggested that the differentiation of e-MDSCs is blocked in an IL-6-dependent manner.

To study the effect of tumor-derived IL-6 on the differentiation of myeloid progenitors, IL-6R-blocking antibody (IL-6R Ab) was applied to inhibit IL-6 signal transduction and the activation of the JAK/STAT3 signaling pathway. IL-6R Ab significantly decreased the percentage of CD11b^+^Gr-1^−^ e-MDSCs (14.70 ± 0.52 vs. 23.60 ± 1.51%, *P* = 0.0051) when compared with CD11b^+^Gr-1^+^ MDSCs (45.27 ± 1.825 vs. 49.37 ± 2.0%, *P* = 0.2094) and dramatically increased the percentage of F4/80^+^ cells (19.63 ± 0.62 vs. 11.90 ± 0.76%, *P* = 0.0014) and MHCII^+^ cells (31.13 ± 1.67 vs. 24.80 ± 1.10%, *P* = 0.0340) (Figure 5C). These results implied that tumor-derived IL-6 blocked the differentiation of the myeloid progenitors and thus promoted the amplification and accumulation of CD11b^+^Gr-1^−^F4/80^−^MHC-II^−^ e-MDSCs.

To determine if CD11b^+^Gr-1^−^F4/80^−^MHC-II^−^ e-MDSCs had the capacity to differentiate into mature neutrophils or monocytes in a tumor-free environment *in vitro*, tumor-derived CD11b^+^Gr-1^−^F4/80^−^MHC-II^−^ e-MDSCs were cultured in a tumor-free complete culture medium supplemented with GM-CSF. Flow cytometry results after GM-CSF stimulation showed that the percentage of CD11b^+^Gr-1^+^ MDSCs (14.23 ± 1.45 vs. 42.47 ± 2.28%, *P* = 0.0005), F4/80^+^ cells (3.83 ± 0.96 vs. 35.00 ± 2.20%, *P* = 0.0002), and MHC-II^+^ cells (13.07 ± 1.10 vs. 48.53 ± 2.80%, *P* = 0.0003) increased significantly after 48 h, whereas the percentage of CD11b^+^Gr-1^−^ MDSCs decreased (78.63 ± 1.03 vs. 54.53 ± 2.99%, *P* = 0.0016). However, no comparable change was observed in the percentage of Ly6G^+^ cells (4.40 ± 0.53 vs. 5.53 ± 0.75%, *P* = 0.2858) (Figure 5D). These results implied that the 4T1-induced blockade of the myeloid progenitor differentiation in CD11b^+^Gr-1^−^ e-MDSCs was reversible. The differentiation capacity was recovered after a deprivation of the tumor-derived factors *in vitro*. In this instance, the CD11b^+^Gr-1^−^ e-MDSCs differentiated into monocytes instead of neutrophils after GM-CSF stimulation.

### SOCS3 Suppression and Sustained Activation of the JAK/STAT Signaling Pathway in e-MDSCs Is IL-6 Dependent

**Figure 6:**
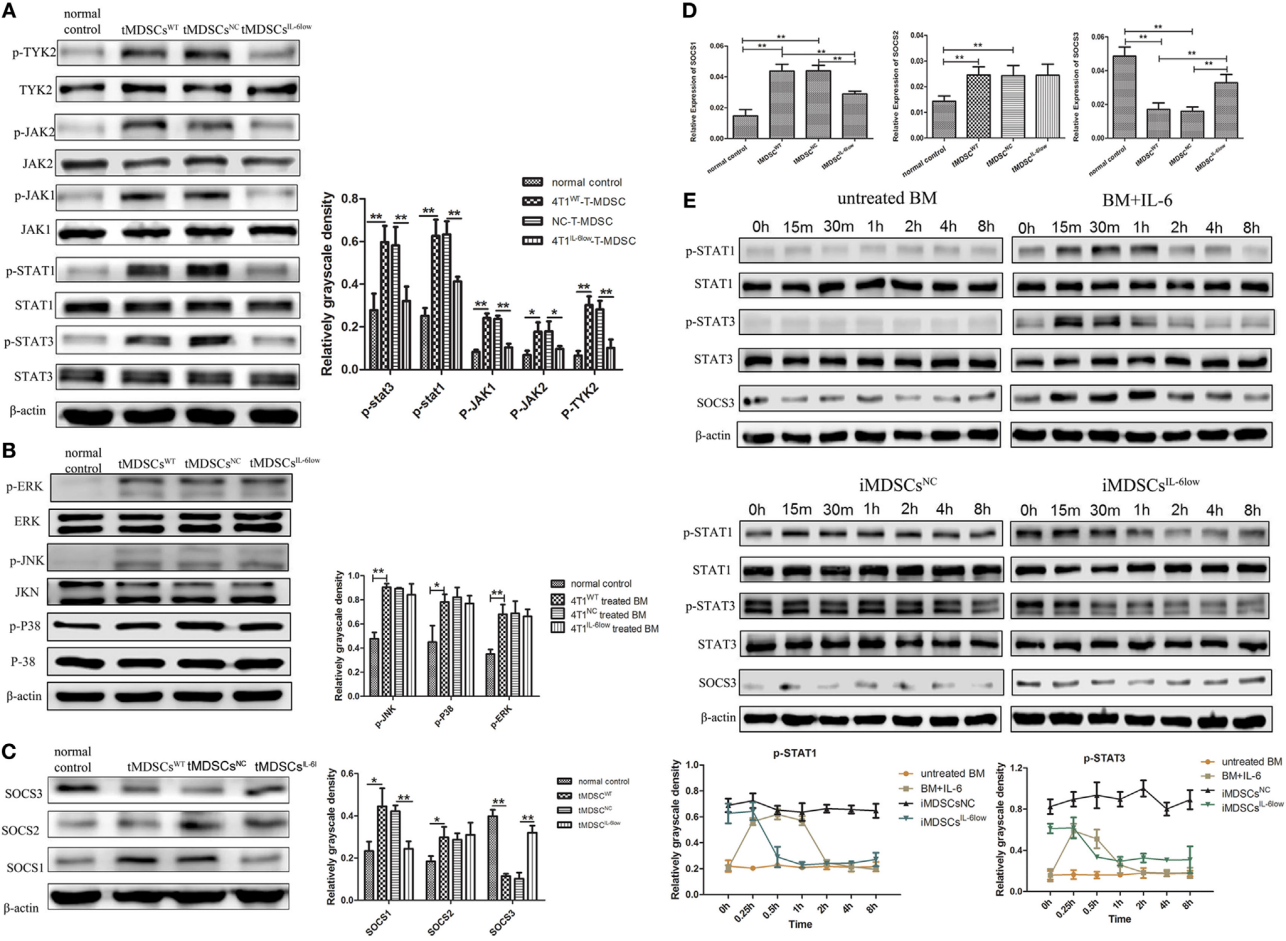
Interleukin-6 (IL-6)-mediated suppressed expression of SOCS3 resulting in the hyperactivation of the JAK/STAT pathway in CD11b^+^Gr-1^−^ early-stage MDSCs (e-MDSCs). Western blotting was performed to detect the JAK/STAT **(A)** and MAPK **(B)** on the whole-cell extracts of primary CD11b^+^Gr-1^−^ e-MDSCs isolated from different IL-6-expressing tumors. **(C)** The protein expression of SOCS1–3 in primary CD11b^+^Gr-1^−^ e-MDSCs. **(D)** The mRNA of SOCS1–3 in primary CD11b^+^Gr-1^−^ e-MDSCs was examined by RT-PCR. **(E)** Bone marrow (BM) cells were treated with 4T1^NC^ and 4T1^IL-6low^ separately to induce iMDSCs^NC^ and iMDSCs^IL-6low^. The activation status of the JAK/STAT pathway downstream of IL-6 signaling in induce MDSCs (iMDSCs) was detected at different time point. BM cells exposed to IL-6 (40 ng/mL) for 15 min were as control. The levels of phosphorylated JAK1 (p-JAK1), p-JAK2, p-TYK2, p-STAT1, p-STAT3, p-P38, p-ERK, and p-JNK were compared using the density ratio of phosphorylated protein to total protein. The levels of the suppressor of cytokine signaling (SOCS) protein were compared using the density ratio of the indicated protein to β-actin (*P < 0.05 and **P < 0.01).

We assessed the expression and phosphorylation of multiple functional proteins induced by IL-6, including JAK/STAT pathway and MAPK signaling pathway using Western blotting. A comparable increase in p-JAK1, p-JAK2, p-TYK2, p-STAT1, and p-STAT3 was detected in tMDSC^WT^ when compared with normal controls (Figure 6A). However, the levels of the phosphorylated proteins mentioned above were reduced significantly in tMDSC^IL-6low^ when compared with those in tMDSC^NC^ (Figure 6A), which implied that the enhanced phosphorylation of the STAT proteins in tMDSC^WT^ is IL-6 dependent. The results about MAPK pathway showed that phosphorylated P38 mitogen-activated protein kinases (p-P38), extracellular regulated protein kinases (p-ERK), and c-Jun N-terminal kinase (p-JNK) increased significantly, though these changes have no relation to IL-6 levels (Figure 6B). All above data showed that the JAK/STAT signaling pathway participated in IL-6-related e-MDSCs accumulation, rather than the MAPK signaling pathway.

Next, we compared the expression of SOCS1–3 in tMDSC^WT^ and normal controls at the mRNA and protein levels since the deficiency of the SOCS proteins has been reported to induce an aberrantly sustained activation of the JAK/STAT signaling pathway in human breast cancer e-MDSCs (9). The results showed that the SOCS1 and SOCS2 mRNA levels increased, but the SOCS3 mRNA level significantly decreased in tMDSC^WT^ when compared with the levels in normal controls. Moreover, the protein levels of SOCS1 decreased and SOCS3 increased in tMDSC^IL-6low^ when compared with the levels in tMDSC^NC^ (Figure 6C). Similar expression patterns of both SOCS1 and SOCS3 were confirmed at the mRNA level (Figure 6D). Therefore, these results implied that tumor-derived IL-6 plays a significant role in the suppression of SOCS3 both at the mRNA and protein levels in tMDSCs, which consequently triggered the sustained activation of the JAK/STAT signaling pathway.

To identify the effect of SOCS3, BM cells were treated with 4T1^NC^ and 4T1^IL-6low^ separately to induce iMDSCs^NC^ and iMDSCs^IL-6low^. Untreated BM cells were exposed to IL-6 (40 ng/mL) for 15 min, and the activation status of the JAK/STAT pathway downstream of IL-6 signaling was detected as control at different time point. In IL-6-stimulated BM cells, the levels of phosphorylated STAT1 and STAT3 proteins were increased at 15 min but decreased at 30 min, disappearing entirely at 2 h. Sustained phosphorylation of STAT1 and STAT3 proteins was observed in iMDSCs ^NC^, which was maintained for a longer time than in normal IL-6-stimulated BM cells (2 vs. 8 h). Meanwhile, the expression of SOCS3 and STAT3 phosphorylation was parallel in IL-6-stimulated BM cells. But in iMDSCs^NC^, SOCS3 expression was decreased significantly and did not response to IL-6 stimulation, which lead to sustained activation of STAT3. In iMDSCs^IL-6low^, phosphorylation levels of STAT1 and STAT3 were reduced at 1 h, and SOCS3 changed along with p-STAT3 (Figure 6E). All above data showed that SOCS3 suppression and sustained activation of STAT3 occurred in e-MDSCs, which were correlated with tumor-derived IL-6. Therefore, the short-term IL-6 simulation induced SOCS3, and SOCS3 in a feedback mechanism shows a negative effect on IL-6 signaling. But long-term IL-6 stimulation in local tumor microenvironment executed significant inhibitory effect on SOCS3 in myeloid cells and thus induced sustained activation of the JAK/STAT pathway.

### IL-6R Ab and STAT3 Antagonist Inhibited Tumor Growth and Metastasis by Attenuating the Accumulation and Functions of e-MDSCs *In Vivo*

**Figure 7:**
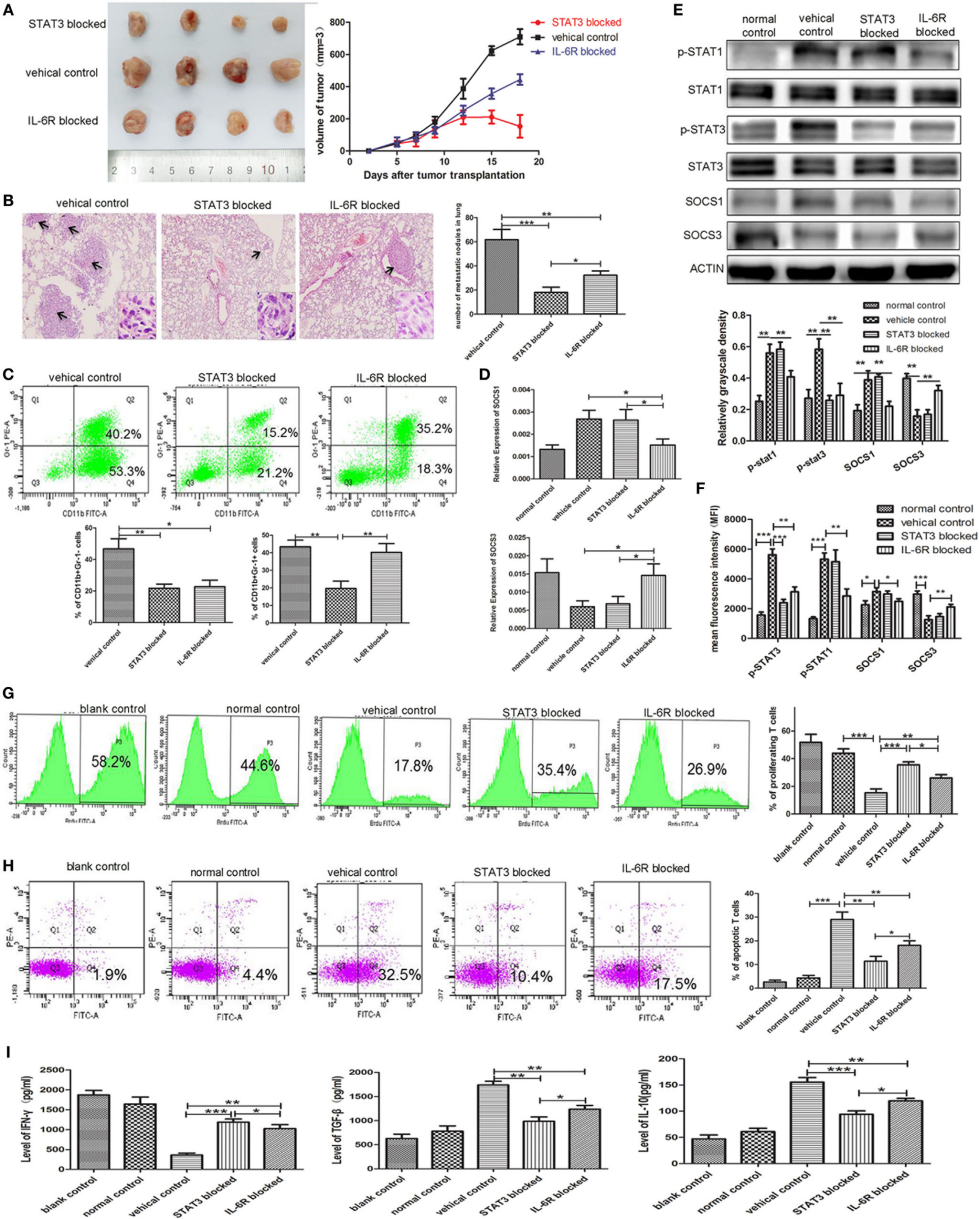
Blocking STAT3 activation or IL-6 receptor (IL-6R) inhibits myeloid-derived suppressor cell (MDSC) development and tumor metastasis in the 4T1 mouse mammary tumor model. The mammary fat pads of BALB/c mice were injected with 4T1 mouse mammary carcinoma cells. After 5 days of tumor implantation, mice were treated intraperitoneally with JSI-124 or IL-6R mAb at for 13 days. **(A)** Tumor sizes in mice were monitored during JSI-124 or IL-6R mAb treatment. On day 18, mice were sacrificed and the tumors and spleens were separated. **(B)** Pulmonary metastatic nodules were counted based on H&E-stained slides. **(C)** Tumor-infiltrating CD45^+^ cells were gated, and MDSCs infiltration in tumors treated with JSI-124 or IL-6R mAb was detected by flow cytometry. **(D)** SOCS1 and SOCS3 in MDSCs treated with JSI-124 or IL-6R Ab were determined by RT-PCR. **(E)** Western blotting was performed to detect the expression of p-STAT3, p-STAT1, SOCS1, and SOCS3 in JSI-124 or IL-6R mAb-treated early-stage MDSCs (e-MDSCs). **(F)** The expression of p-STAT3, p-STAT1, SOCS1, and SOCS3 in JSI-124 or IL-6R mAb-treated e-MDSCs was confirmed by intracellular flow cytometry. And the expression levels were presented by mean fluorescence intensity. JSI-124 and IL-6R mAb-treated CD11b^+^Gr-1^−^ e-MDSCs were isolated from tumors and cocultured with T cells. T cell proliferation **(G)** and apoptosis **(H)** were determined by flow cytometry. **(I)** Secreted cytokines by T cells were determined by enzyme-linked immunosorbent assay (*P < 0.05; **P < 0.01; and ***P < 0.001).

IL-6 receptor Ab and STAT3 antagonist JSI-124 were used to inhibit the IL-6-induced activation of the JAK/STAT signaling pathway. In addition, we found that both significantly inhibited the growth of tumors *in vivo* (*P* < 0.001, *P* < 0.001, Figure 7A) and reduced the number and size of metastatic lung nodules (32.33 ± 2.028 vs. 18.00 ± 2.52 vs. 55.00 ± 3.61, *P* = 0.0054, *P* = 0.0011, Figure 7B). Simultaneously, significant decrease in the number of CD11b^+^Gr-1^−^ e-MDSCs in primary tumor tissues was also observed in IL-6R Ab- and JSI-124-treated mice when compared with untreated controls (22.73 ± 2.39 vs. 21.70 ± 1.55 vs. 46.70 ± 3.70%, *P* = 0.0055, *P* = 0.0034, Figure 7C).

We isolated primary e-MDSCs from the IL-6R Ab- and JSI-124-treated mice and found that the p-STAT3 protein levels in both groups significantly decreased when compared with the cells isolated from untreated controls (Figure 7D). Moreover, the mRNA and protein levels of SOCS1 decreased while those of SOCS3 increased in IL-6R Ab-treated e-MDSCs. However, no significant difference in the expressions of SOCS1 and SOCS3 was observed in JSI-124-treated e-MDSCs (Figures 7D,E). Furthermore, the STAT and SOCS expression in e-MDSCs were also determined using intracellular flow cytometry, and mean fluorescence intensity was utilized to present the expression quantity. The results were consistent the WB results (Figure 7F). Above data implied that tumor-derived IL-6 predominantly triggered SOCS3 suppression in e-MDSCs, and hence it could be reversed by using the IL-6R blocking antibody.

Furthermore, normal spleen T cells were cocultured with either JSI-124- or IL-6R Ab-treated e-MDSCs *in vitro* to study their effects on the proliferation, apoptosis, and cytokine production of T cells. Both IL-6R Ab- and JSI-124-treated e-MDSCs induced more proliferation (26.10 ± 1.39 vs. 35.60 ± 1.22 vs. 15.37 ± 1.58%, *P* = 0.0070, *P* = 0.0005, Figure 7G), but lesser apoptosis of T cells (18.07 ± 1.15 vs. 11.40 ± 1.19 vs. 28.97 ± 1.84%, *P* = 0.0074, *P* = 0.0013, Figure 7H). Accordingly, the secretion of IFN-γ (1,024 ± 57.39 vs. 1,187 ± 45.15 vs. 360.90 ± 27.41 pg/mL, *P* = 0.0034, *P* = 0.0006) was higher, but the secretion of IL-10 (94.05 ± 3.743 vs. 119.7 ± 2.85 vs. 155.6 ± 5.02 pg/mL, *P* = 0.0034, *P* = 0.0006) and TGF-β (1,237 ± 45.70 vs. 985.5 ± 52.45 vs. 1,740 ± 44.01 pg/mL, *P* = 0.0014, *P* = 0.0004) was lower in T cells stimulated by either IL-6R Ab- or JSI-124-treated e-MDSCs when compared with the levels in T cells stimulated by untreated e-MDSCs (Figure 7I). These results implied that blocking the IL-6 signal or inhibiting the activation of the JAK/STAT signaling pathway could dramatically reduce tumor growth and metastasis by attenuating the accumulation and immunosuppressive capacity of e-MDSCs *in vivo*.

## Discussion

Recent studies have demonstrated that the IL-6 plays an important role in tumor progression and metastasis (28) and is expressed in approximately 50% of breast cancers. IL-6 also correlated with poor prognosis and advanced disease in breast cancer patients (29). But the direct effect of IL-6 on breast cancer cell is not fully understood, and recent studies were paradoxical on IL-6 dependent effect on the proliferation and invasion of breast cancer cells. Studies showed both direct growth inhibitory effects (30–32), while other studies show growth promoting effects (33, 34) or no effects at all (35, 36). Asgeirsson et al. have demonstrated that IL-6 decreased cell adhesion of breast cancer cell lines T47-D, ZR-75-1, and MCF-7 to promote metastasis, but the effect was not observed in MDA-MB-231 (37). Ectopic stable IL-6 expressing MCF-7 breast adenocarcinoma cells exhibited an EMT phenotype characterized by impaired E-cadherin expression and induction of Vimentin, N-cadherin, Snail, and Twist (38). And researchers proposed that a pattern emerges from above studies, showing that ER-positive breast cancer cells are receptive to exogenous IL-6, while ER-negative breast cancer cells are mostly unresponsive, probably due to a high autocrine production of IL-6 in ER-negative breast cancer cells (39). And in present study, our results showed that IL-6 knockdown has no effect on 4T1 cells. And merely reducing the level of IL-6, rather than fully blocking the interaction between IL-6 and IL-6R, could not affect the downstream JAK/STAT signaling pathway and the proliferation and invasion of 4T1 cells *in vitro*. However, IL-6 knockdown exclusively displayed significant inhibition on 4T1 tumor growth and metastasis *in vivo* which indicated that microenvironment plays a vital role in breast cancer development and progression.

Recent evidence showed that IL-6 played critical roles in cancer development and progression by regulating the tumor microenvironment (40), which threw new light on the molecular mechanism regulating the IL-6-induced growth and metastasis of mammary carcinoma *in vivo*. Several studies have confirmed the relationship between tumor-derived IL-6 and MDSCs in a wide variety of tumor types such as esophageal cancer, prostate cancer, and hepatocellular carcinoma (11, 41, 42). In breast cancers, IL-6-expressing 4T1 mammary tumor-bearing mice were reported to dramatically enhance the recruitment of CD11b^+^Gr-1^+^ MDSCs in the spleen, primary tumor mass, and metastasizing organs when compared with low IL-6-expressing EMT6 mice (13). Consistently, we observed the enhanced accumulation of CD11b^+^Gr-1^+^ MDSCs in the spleens and tumors of 4T1^WT^ mice in our study. Furthermore, we found a significant increase in the immature MDSC subset with the CD11b^+^Gr-1^−^F4/80^−^MHC-II^−^ phenotype in the primary tumors of the 4T1^WT^ mice, which were regulated by tumor-derived IL-6. These immature MDSCs displayed a higher positive correlation with primary tumor size and the number of lung metastatic nodules in tumor-bearing mice than CD11b^+^Gr-1^+^ MDSCs, which is a major MDSC subset in primary tumors and more responsible for IL-6-stimulated tumor growth and metastasis.

Recently, a more systemic declaration of the characteristic phenotypes of different MDSC subsets was recommended, in which MDSCs were divided into three subsets in humans: PMN-MDSCs, MO-MDSCs, and e-MDSCs (6). In humans, the distinct phenotypes of PMN-MDSCs and M-MDSCs are CD11b^+^HLA-DR^−/lo^CD14^−^CD15^+^ and CD11b^+^HLA-DR^−/lo^CD14^+^CD15^−^, respectively. Accordingly, the mouse equivalents of PMN-MDSCs and MO-MDSCs have the phenotypes of CD11b^+^Ly6G^+^Ly6C^lo^ and CD11b^+^Ly6G^+^Ly6C^hi^, respectively. As mentioned by Bronte et al., e-MDSCs or early-stage MDSCs comprise more immature progenitors than conventional MDSCs, which were known as Lin^−^HLA-DR^−^CD33^+^ (6). The mouse equivalent of e-MDSCs has not yet been identified (6). In our previous study, we defined a poorly differentiated e-MDSC subset in human breast cancer with the phenotype of Lin^−^HLA-DR^−^CD45^+^CD33^+^CD13^+^CD14^−^CD15^−^ (7). Similarly, we found that the CD11b^+^Gr-1^−^F4/80^−^MHC-II^−^ MDSCs in murine primary mammary tumors consisted of more myeloid progenitors and exerted potent suppressive effects on T cell immunity. More detailed examination of the lineage potentials of CD11b^+^Gr-1^−^F4/80^−^MHC-II^−^ MDSCs confirmed that they skewed toward monocyte formation instead of neutrophil formation after GM-CSF stimulation. These results implied that CD11b^+^Gr-1^−^F4/80^−^MHC-II^−^ MDSCs represented the equivalent of the e-MDSC subset in mice.

In this study, we found that tumor cells blocked the differentiation of myeloid progenitors in CD11b^+^Gr-1^−^ e-MDSCs, but their capacity to differentiate was restored after a deprivation of tumor-derived factors. Consistently, a previous study reported that PMN-MDSCs could resemble neutrophils phenotypically and functionally after being stimulated by GM-CSF *in vitro* (43). After culturing tumor-derived MDSCs in the absence of tumor-derived factors or transferring MDSCs to tumor-free recipients, more mature macrophages and DCs were generated (44, 45). All these reports showed that tumor-derived factors abrogated the differentiation of myeloid cells. Furthermore, we induced e-MDSCs by coculturing murine BM cells with 4T1 mammary cancer cells *in vitro* and found that the differentiation toward mature F4/80^+^ or MHCII^+^ cells was obviously blocked upon GM-CSF stimulation. Blocking the IL-6 signaling pathway reversed the differentiation block and promoted the generation of F4/80^+^ or MHC-II^+^ mature myeloid cells. A similar phenomenon was observed in primary tumors in which 70% of CD11b^+^Gr-1^−^ MDSCs were F4/80^−^ and MHC-II^−^ immature myeloid cells. Knockdown tumor-derived IL-6 increased the percentage of mature myeloid cells significantly. All these findings suggested that tumor-derived IL-6 was the critical factor that blocked myeloid differentiation and the development of e-MDSCs.

It has been reported that the status of differentiation of myeloid progenitors partly influenced the immunosuppressive capacity of MDSCs. Pu et al. identified that both granulocyte/macrophage progenitors and common myeloid progenitors, freshly isolated from tumor-bearing animals, were capable of inhibiting polyclonal stimuli- and alloantigen-induced T cell proliferation. Strikingly, early-stage myeloid progenitors displayed much stronger suppressive capacities than conventional MDSCs (46). Zhou et al. reported that CD115^+^Ly6C^−^ MDSCs were more immature and showed higher suppressive activities than CD115^+^Ly6C^+^ MDSCs (47). Another previous study showed that MDSCs with higher Gr-1 expression were more mature, CD11b^+^Gr-1^low^ cells were the most immunosuppressive, CD11b^+^Gr-1^int^ cells were less immunosuppressive, and CD11b^+^Gr-1^high^ cells (mostly granulocytes) were the least immunosuppressive (48). Consistent with these studies, we found that CD11b^+^Gr-1^−^ e-MDSCs isolated from primary 4T1 tumors were more immature but exerted more potent immunosuppressive capacities than conventional CD11b^+^Gr-1^+^ MDSCs. The robust suppression of T cells was recovered when IL-6R Ab or JSI-124 was administrated, which implied that tumor-derived IL-6 was the key trigger that regulated the development of competent e-MDSCs.

Interleukin-6 is a well-known trigger that activates the JAK/STAT signaling pathway, which plays crucial roles in the amplification and function of MDSCs in multiple tumors (49, 50). Interestingly, we found an aberrantly activated JAK/STAT signaling pathway during the IL-6-initiated development of the CD11b^+^Gr-1^−^ e-MDSCs in this study, which demonstrated significant upregulation of the phosphorylated STAT1, STAT3, JAK1, JAK2, and TYK2 proteins. After inhibiting the phosphorylation of STAT3 *via* the specific antagonist JSI-124, a considerable decrease in the suppression of T cell immunity was confirmed. A consistent phenomenon was observed in human breast cancer in which the sustained activation of the JAK/STAT signaling pathway dominated the amplification and immunosuppressive capacity of the Lin^−^HLA-DR^−^CD45^+^CD13^+^CD33^+^CD14^−^CD15^−^ e-MDSCs (8, 9). The results in both human and mice implied that tumor-derived IL-6 involved in the hyperactivation of the JAK/STAT signaling pathway, which was the main cause of the development of competent e-MDSCs in breast cancer.

The studies about MDSCs in prostate cancer demonstrated that SOCS3 negatively regulated the development and function of MDSCs by inhibiting STAT3 activation. Moreover, SOCS3-deficient mice elevated Gr-1^+^CD11b^+^ MDSCs in tumors and exhibited heightened STAT3 activation (23). Besides, we previously demonstrated that significant suppression of SOCS3 in human e-MDSCs induced persistent activation of the JAK/STAT signaling pathway and expression of downstream functional genes, such as IDO, IL-10, and TGF-β, which produce immunosuppressive microenvironments locally (8, 9). In this study, significant suppression of SOCS3, both at the mRNA and protein levels, was identified in primary e-MDSCs isolated from 4T1 xenografts and induced e-MDSCs by coculturing murine BM cells with 4T1 cells. All above results suggested that SOCS3 plays critical role in MDSC development. And in this study, IL-6R Ab effectively reversed SOCS3 suppression, recovered T cell immunity, and abolished IL-6-related differentiation block of myeloid cells *in vitro and vivo*. Consistently, chemokine receptor 5 (CCR5) blockade inhibited IL-6-STAT3 pathway *via* SOCS3, and anti-CCR5 antibody treatment could inhibit the growth of tumor *via* upregulated SOCS3 to decrease MDSCs accumulation and immunosuppressive capacity *in vivo* (51). Therefore, it may be a practical scheme to upregulate the SOCS3 expression to inhibit MDSCs accumulation and reverse immunosuppression.

This study showed that IL-6 alone showed less effect on e-MDSCs development compared with 4T1 supernatant or 4T1 cells. The remarkable difference between IL-6- and high IL-6-expressing tumor microenvironment suggested that it was the IL-6-related tumor-derived factors, rather than IL-6 directly, that influenced e-MDSCs development. Besides, IL-6 was strong inducer of SOCS3 expression *via* STAT3 phosphorylation (52). But surprisingly, we found that e-MDSCs exposed in high IL-6 expressing tumor microenvironment presented SOCS3 suppression and sustained activation of JAK/STAT, while IL-6 short stimulation caused temporary SOCS3 regulation and JAK/STAT activation. The quite the opposite effect of rIL-6 and tumor microenvironment with high IL-6 on both e-MDSCs accumulation and SOCS3 expression also suggested that IL-6-related tumor-derived factors, but not IL-6 directly influenced SOCS3 suppression. It has been reported that promoter methylation and microRNAs were two main mechanisms of SOCS3 suppression. The CpG island in the human SOCS3 genomic sequence was identified, which encompasses the promoter and coding sequence of human SOCS3 gene (53). And hypermethylation of SOCS3 promoter region was observed in H460 cells (54). miRNAs are about 23 nucleotides long, single-stranded molecules and regulate gene expression controlling various biological processes. Researchers reported that many miRNAs participated in the regulation of SOCS3 expression, such as miRNA-127, miRNA-124a, miR-122, and miR-483-5p (55–58). It is reported that increased miR-30a-mediated SOCS3 decrease was involved in MDSC function and differentiation, and miR-30a antagomir could inhibit the growth of tumor *via* upregulated SOCS3 to decrease MDSCs accumulation and immunosuppressive capacity *in vivo* (59). Therefore, further study on the transcriptional and post-transcriptional regulatory mechanisms involved in IL-6-related SOCS3 suppression will be conducted.

The fact that MDSCs play an important role in the regulation of tumor growth has stimulated the search for potential target therapeutic strategies (60). In this study, we confirmed the efficacy of target therapy against IL-6 signaling and STAT3 phosphorylation in the treatment of breast cancer. IL-6R Ab and a specific STAT3 antagonist inhibited the growth and metastasis of IL-6^+^ mammary cancer *in vivo* and dramatically reduced the accumulation of e-MDSCs. Recently, tocilizumab, a humanized recombinant mAb against the IL-6R, showed the potential to improve prognosis in IL-6-expressing lung cancer and recurrent epithelial ovarian cancer (61, 62). Our finding is consistent with a previous report on MR16-1, a rodent analog of tocilizumab and a commercial humanized recombinant IL-6R mAb, that enhanced anti-tumor activity by eliminating MDSCs (63). STAT3 antagonist JSI-124 suppressed both CD11b^+^Gr-1^−^ e-MDSCs and conventional CD11b^+^Gr-1^+^ MDSCs simultaneously and induced considerable recovery of T cell immunity *in vivo* and *in vitro*, thereby displaying more significant anti-tumor efficiency than IL-6R Ab. Furthermore, another previous study demonstrated that the STAT3 antagonist JSI-124 induced MDSCs to differentiate into immunogenic DCs (64). However, considering that the STAT3 antagonist JSI-124 has more severely toxic adverse effects and is limited in animal experiment. IL-6R Ab might be a more promising therapeutic strategy for breast cancer.

In conclusion, this study provided new insights into investigating the crosstalk between breast cancer cells and regulatory immunocytes in tumor microenvironments. In murine mammary carcinoma, tumor-derived IL-6 impaired the differentiation of myeloid progenitors, promoted the development of CD11b^+^Gr-1^−^F4/80^−^MHC-II^−^ e-MDSCs, and potently suppressed T cell immunity, in which IL-6-dependent SOCS3 suppression and the consequential activation of the JAK/STAT signaling pathway are the most crucial molecular events. Targeting the IL-6/STAT3 signaling pathway can significantly restore the differentiation of myeloid progenitors and reverse the immunosuppressive capacity of e-MDSCs. Therefore, blocking the IL-6 signaling pathway might be a promising therapeutic strategy for breast cancer treatment.

## Ethics Statement

The experiment was approved by the Ethics Committee for Animal Experiments at the Tianjin Medical University Cancer Hospital and Institute and was performed in accordance with the Guide for the Care and Use of Laboratory Animals.

## Author Contributions

Experiments design: JY, RZ, and WZ; experimental operation: WZ and MJ; experimental instructor: YY, PL, and JC; data analysis: JY, WZ, and WY; article writing: WZ; paper modification: JY.

## Conflict of Interest Statement

The authors declare that the research was conducted in the absence of any commercial or financial relationships that could be construed as a potential conflict of interest. The reviewer ZC and handling Editor declared their shared affiliation.
