# Supplementary material for: SOCS3 Suppression Promoted the Recruitment of CD11b+Gr-1−F4/80−MHCII− Early-Stage Myeloid-Derived Suppressor Cells and Accelerated Interleukin-6-Related Tumor Invasion via Affecting Myeloid Differentiation in Breast Cancer
Source: Front Immunol. 2018 Jul 23;9:1699. doi: 10.3389/fimmu.2018.01699 (PMC6064721; doi:10.3389/fimmu.2018.01699)
Supplement: Supplementary file 1 [file table_1.docx]

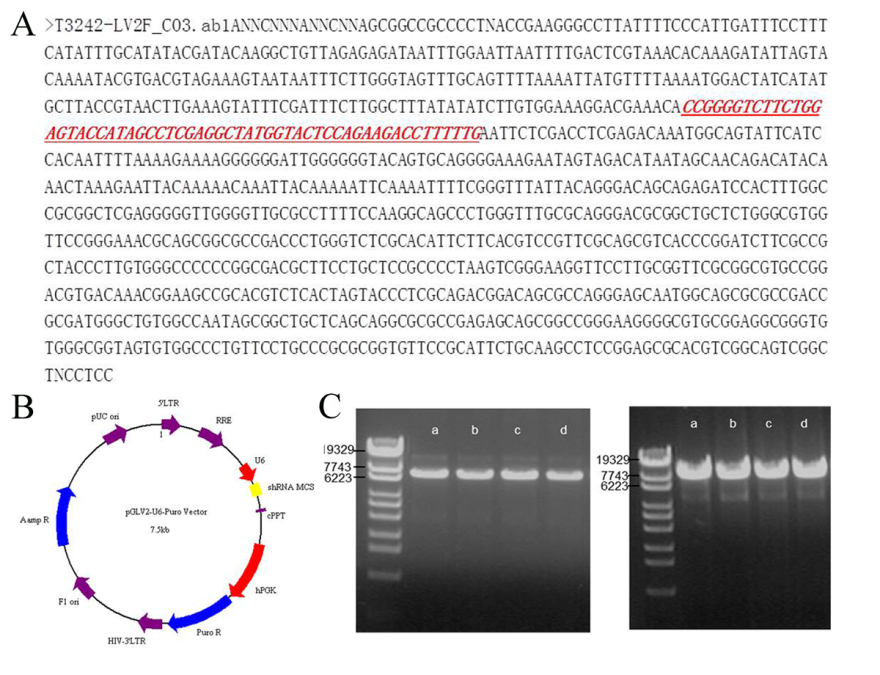


**Supplemental data 1.** Construction of genetically modified breast cancer cell lines. IL-6-specific interference sequence (5'-GGTCTTCTGGAGTACCATAGC-3') was inserted into pGLV2/U6/Puro vectors by digesting them with BshTI and EcoRI restriction enzymes (Thermo Fisher Scientific, USA). The correct recombinant plasmids were confirmed via DNA sequencing and transfected into 293T cells to produce lentiviral particles as indicated. (A) Sequencing result of constructed plasmid. (B) Ideograph of constructed plasmid. (C) Plasmids and digested plasmids with Lambda DNA/Eco130I were confirmed using DNA gel electrophoresis.
