# Supplementary material for: SOCS3 Suppression Promoted the Recruitment of CD11b+Gr-1−F4/80−MHCII− Early-Stage Myeloid-Derived Suppressor Cells and Accelerated Interleukin-6-Related Tumor Invasion via Affecting Myeloid Differentiation in Breast Cancer
Source: Front Immunol. 2018 Jul 23;9:1699. doi: 10.3389/fimmu.2018.01699 (PMC6064721; doi:10.3389/fimmu.2018.01699)
Supplement: Supplementary file 2 [file table_2.docx]

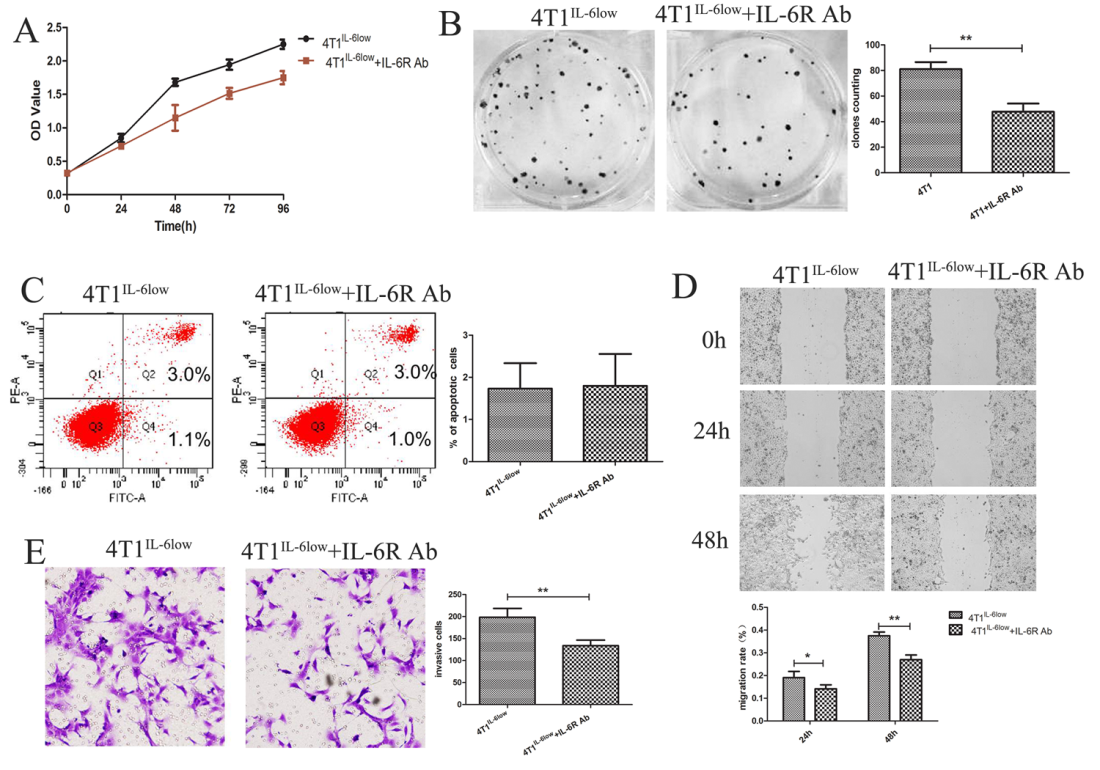


**Supplemental data 2**. The effect of IL-6R blocking on 4T1^IL-6low^ cells. IL-6R antibody (10 ug/ml) was utilized to block IL-6 signaling pathway in 4T1^IL-6low^ cells. (A) Cell growth was monitored by CCK-8 assay (B) Clonogenic survival analysis of infected 4T1^IL-6low^ cells. (C) The quantification of the infected 4T1^IL-6low^ cell apoptosis was detected using the Annexin V-FITC apoptosis detection kit. Apoptotic cells were recognized by Annexin V^+^PI^-^. (D) Wound healing assay was used to study the migratory ability of infected 4T1^IL-6low^ cells (original magnification × 40). (E) The infiltrative and metastatic capability of infected 4T1 cells was evaluated by invasion assay (original magnification × 10). The stained cells from 5 selected views were observed under a light microscope at 200× magnification. *, P < 0.05; ** P < 0.01; ***, P < 0.001.
