# Supplementary material for: SOCS3 Suppression Promoted the Recruitment of CD11b+Gr-1−F4/80−MHCII− Early-Stage Myeloid-Derived Suppressor Cells and Accelerated Interleukin-6-Related Tumor Invasion via Affecting Myeloid Differentiation in Breast Cancer
Source: Front Immunol. 2018 Jul 23;9:1699. doi: 10.3389/fimmu.2018.01699 (PMC6064721; doi:10.3389/fimmu.2018.01699)
Supplement: Supplementary file 3 [file table_3.docx]

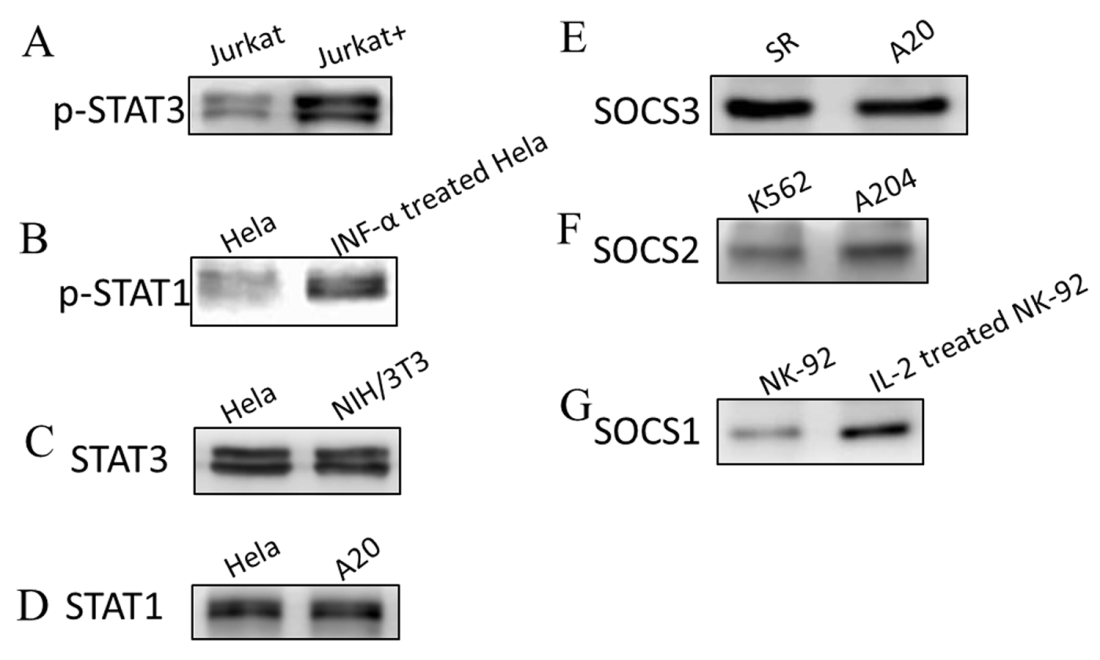


**Supplemental data 3**. Validity of STAT and SOCS antibody. According to the antibody operation manual, (A) the p-STAT3 in extracts from IFN-α treated Jurkat cells using the Phospho-Stat3 (Tyr705) (D3A7) XP® Rabbit mAb (1:1000, CST), (B) p-STAT1 in extracts from IFN-α treated Hela cells. And we can see phosphorylated STAT3 and STAT1 increased after cytokines stimulation. (C) STAT3 in Hela and NIH/3T3 cell lysates was detected using Stat3 (124H6) Mouse mAb (1:1000, CST). (D) STAT1 in extracts of Hela and A20 cells were determined using Stat1 (D1K9Y) Rabbit mAb (1:1000, CST). Besides, SOCS3 in SR and A20 cell lysates(E), SOCS2 in extracts of K562 and A204 cells (F), and SOCS1 in IL-2 stimulated NK-92 cells lysate (G) were determined using SOCS3 (L210) Antibody (1:1000, CST), SOCS2 Antibody (1:1000, CST), and SOCS1 (A156) Antibody (1:1000, CST) respectively.
