# Supplementary material for: SOCS3 Suppression Promoted the Recruitment of CD11b+Gr-1−F4/80−MHCII− Early-Stage Myeloid-Derived Suppressor Cells and Accelerated Interleukin-6-Related Tumor Invasion via Affecting Myeloid Differentiation in Breast Cancer
Source: Front Immunol. 2018 Jul 23;9:1699. doi: 10.3389/fimmu.2018.01699 (PMC6064721; doi:10.3389/fimmu.2018.01699)
Supplement: Supplementary file 4 [file table_4.docx]

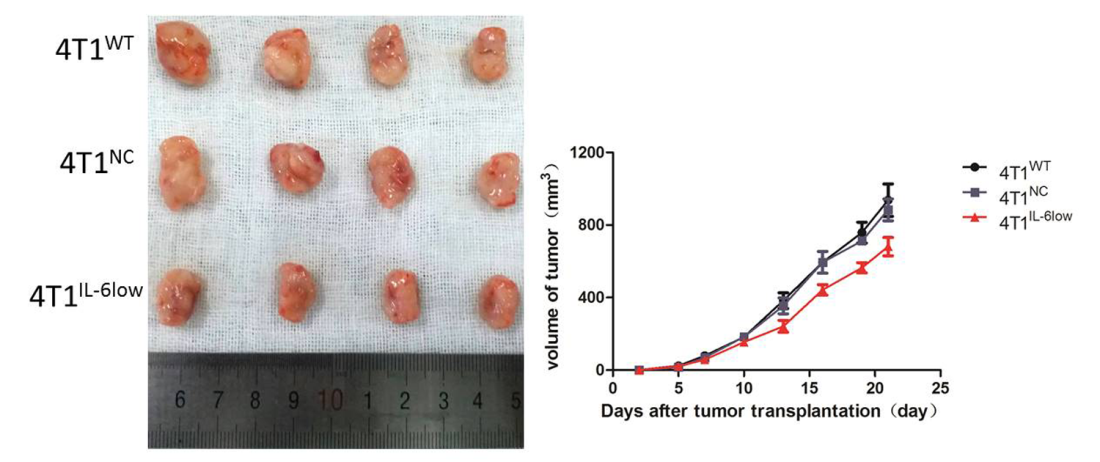


**Supplemental data 4.** The effect of IL-6 knockdown on tumor growth in NOD/SCID mice. 4T1^WT^, 4T1^NC^, and 4T1^IL-6low^ were injected into the mammary fat pads of female NOD/SCID mice. Tumor sizes were monitored and calculated using the following equation: V = ((π × a × b^2^)/6), where a is the length and b is the width of the tumor. Mice were sacrificed on day 21, and tumors were separated.
